# Luminescence Thermometry via Multiparameter Sensing in YV_1–*x*_P*_*x*_*O_4_:Eu^3+^, Er^3+^

**DOI:** 10.1021/jacs.5c02306

**Published:** 2025-04-04

**Authors:** Yixuan Ma, Xiaopeng Zhou, Jiapeng Wu, Zhijie Dong, Lizhi Cui, Yuhua Wang, Andries Meijerink

**Affiliations:** † National and Local Joint Engineering Laboratory for Optical Conversion Materials and Technology of National Development and Reform Commission, School of Materials and Energy, Lanzhou University, Lanzhou 730000, China; ‡ Condensed Matter and Interfaces, Debye Institute for Nanomaterials Science, Utrecht University, Princetonplein 1, 3584CC Utrecht, The Netherlands

## Abstract

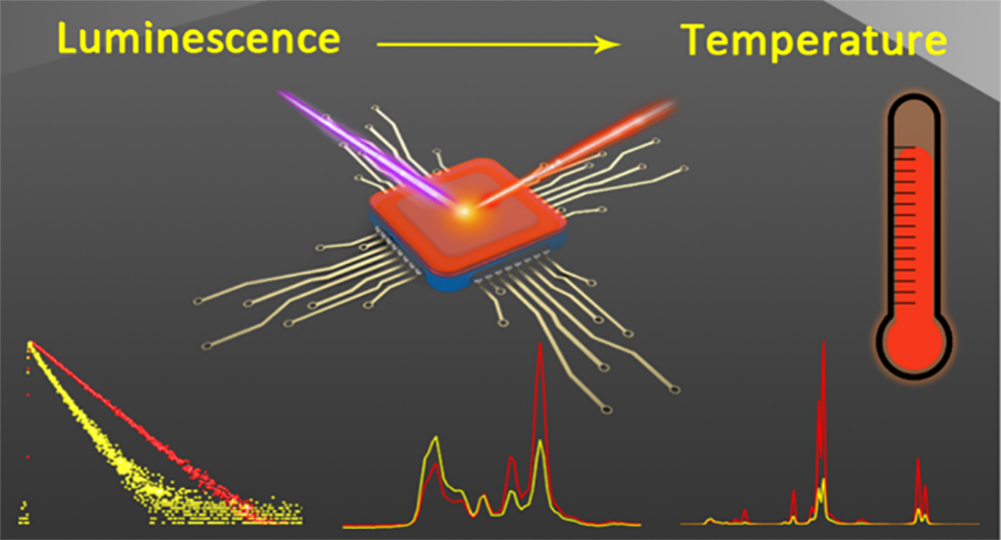

Luminescence thermometry is a remote temperature sensing technique that utilizes temperature-dependent luminescence properties. Lanthanide-doped materials with two thermally coupled emitting levels displaying a variation in luminescence intensity ratio (LIR) with temperature have been successfully explored to design sensitive luminescent thermometers. However, the low absorption strength of lanthanide parity-forbidden 4f^*n*^ → 4f^*n*^ transitions reduces the brightness. Also, this Boltzmann-type thermometer is only sensitive within a limited temperature range. To address these issues, we report here YV_1–*x*_P*_*x*_*O_4_:Eu^3+^, Er^3+^ as a luminescent thermometer. This material utilizes the sensitized emission of Ln^3+^ by strong and broad vanadate charge transfer absorption and has a wide and tunable optimum temperature range by controlling the thermal quenching of Eu^3+^ emission through a variation of *x*. The new temperature probe offers a single material with multiple temperature-dependent luminescence properties, viz. the LIR of ^2^H_11/2_/^4^S_3/2_ emission of Er^3+^, the LIR of the integrated Er^3+^ and Eu^3+^ emission intensities, and the Eu^3+^ emission lifetime. Both micro- and nanocrystalline temperature probes are reported to achieve relative sensitivities (*S*_r_) from ∼0.5%/K to over 5%/K in a wide temperature range of 300–873 K. To demonstrate practical applicability, the luminescent thermometer was applied to in situ chip temperature detection revealing temperature accuracies better than 1 K.

## Introduction

Temperature sensing has universal importance and is extremely diverse, varying from common applications such as recording fevers and temperature in refrigerators to controlling chemical reactions in industrial plants and monitoring temperatures in electronic devices. Remote temperature sensing relying on temperature-dependent lanthanide luminescence is rapidly developing with a need for high sensitivity and temperature accuracy.^1−3^ Especially, luminescence nanothermometry is widely applied in biological systems in the 300–350 K temperature range, often using the green upconversion emission of Er^3+^ in NaYF_4_ nanocrystals codoped with Yb^3+^ and Er^3+^.^4−9^ More recently, there has been an effort to develop new temperature probes for remote sensing and high-resolution mapping of temperature distributions at higher temperatures (*T* > 400 K) in fields such as catalysis, microfluidics, and electronic devices.^10−13^

It is important to obtain stable and sensitive temperature detection over a wide temperature range. Luminescence temperature sensing relying on the variation of the emission intensity ratio from two thermally coupled levels, as in the model system NaYF_4_:Er^3+^,Yb^3+^, has many advantages, such as high relative sensitivity *S*_r_, self-referencing, and being largely (but not fully!) insensitive to changes in local surroundings.^14−18^ However, the temperature range with high *S*_r_ is limited and falls off rapidly toward higher temperatures, making these probes less attractive for applications where a wide temperature range needs to be monitored.^19−21^ For many lanthanide-based Boltzmann thermometers, excitation involves parity-forbidden 4f^*n*^ → 4f^*n*^ transitions.^4,11,22,23^ The weak absorption limits the brightness of the nanoprobe, as the brightness is the product of the quantum yield and the absorption strength. Especially in applications benefiting from short acquisition times (such as high-resolution temperature mapping) or with low excitation intensity or emission collection efficiency, high brightness (nano)particles are essential to obtain the desired temperature accuracy.

Here, we report a new luminescent thermometer, YV_1–*x*_P*_*x*_*O_4_ codoped with Eu^3+^ and Er^3+^. In this material, to mitigate the weak absorption of lanthanide ions, sensitization by the vanadate ions is used. The vanadate charge transfer absorption band is a well-known sensitizer for Ln^3+^ emission, and back in 1964, YVO_4_:Eu^3+^ was the first commercially applied Ln-doped phosphor and also relied on this principle.^24,25^ Interestingly, in spite of a long history of research, the energy transfer mechanism remains elusive.^25−27^ It is evident that thermally activated energy migration between vanadate groups is involved; however, the mechanism for the final vanadate to (nearest neighbor) Ln^3+^ transfer step is not clear but has been shown not to involve only dipole–dipole interaction.^27^ The use of YVO_4_ (and also GdVO_4_ or LaVO_4_) as a host for lanthanide ions to design luminescence temperature sensing materials has been explored before,^28−33^ sometimes also using sensitization of Ln^3+^ luminescence by the vanadate group.

The present approach relies on codoping both Er^3+^ and Eu^3+^ in hosts with a mixed composition YV_1–*x*_P*_*x*_*O_4_ (with *x* = 0, 0.25, 0.50, 0.75, and 1) and uses the temperature-dependent emission intensity ratio of different emission lines (^2^H_11/2_/^4^S_3/2_ of Er^3+^ and integrated Er^3+^/integrated Eu^3+^) to accurately determine the temperature over a wide range. In addition, the temperature dependence of the Eu^3+^ emission lifetime is utilized. With varying *x*, the vanadate absorption shifts to lower energy for increasing vanadate content.^34,35^ Here, we show that this allows tuning of the quenching temperature *T*_Q_ for the Eu^3+^ emission, and thus the temperature range where the Er^3+^/Eu^3+^ intensity ratio, as well as the Eu^3+^ emission lifetime, changes. Interestingly, YVO_4_:Eu^3+^ is well-known as a phosphor with a high *T*_Q_ for the Eu^3+^ emission, enabling its application as a color converter in the hot environment of high pressure vapor lamps to obtain a warmer white emission spectrum.^24,36^ However, as far as we know, there are no reports on the quenching temperature or the shift of *T*_Q_ upon changing the V/P ratio. In YV_1–*x*_P_*x*_O_4_:Eu^3+^, we report here an increase from 788 K in YVO_4_ to over 900 K for Y(V_0.25_P_0.75_)O_4_. Based on the three temperature-dependent parameters, accurate temperature sensing with *S*_r_ values of ∼0.5%/K to even ∼9%/K at 873 K is realized. This enables our YV_1–*x*_P_*x*_O_4_:Er^3+^,Eu^3+^ thermometer to achieve optimal temperature sensing over a wide temperature range (RT–873 K). The performance of both nano- and microcrystalline temperature probes was verified under realistic operating conditions by encapsulation onto the surface of the RT9955 chip, revealing temperature accuracies of 1 K or better over a temperature range of 300–500 K, which confirmed that micro- and nanocrystalline Y(P,V)O_4_:Eu^3+^, Er^3+^ is a versatile material for accurate remote temperature sensing.

## Results and Discussion

### Synthesis and Characterization

YV_1–*x*_P_*x*_O_4_:0.5%Eu^3+^ and YV_1–*x*_P_*x*_O_4_:0.5%Eu^3+^, 2%Er^3+^ (*x* = 0, 0.25, 0.5, 0.75 and 1) microcrystals were obtained via a high-temperature solid-state synthesis method,^37−39^ as described in the Supporting Information and shown in Figure S1. X-ray powder diffraction (XRD) characterization shows that for all materials, the diffractograms match well with the standard reference card, which indicates that both P substitution and doping ions Eu^3+^ and Er^3+^ have successfully entered the matrix lattice without generating impurity phases. High-purity single-phase microcrystals were prepared. With the increase of P substitution amount, the diffraction peaks gradually moved to larger angles, which is due to the fact that the ionic radius of P^5+^ is smaller than V^5+^.^40^ To verify the incorporation of Eu^3+^ and Er^3+^, as well as the ratio of VO_4_^3–^ vs PO_4_^3–^, after synthesis, the ICP-OES elemental analysis was performed. The results in Table S5 show that the actual compositions are very close to the elemental ratios of the starting materials. SEM images combined with elemental mapping using EDX (Figure S11) show that the elemental distribution is homogeneous in the solid solutions of various Y(P,V)O_4_:Eu^3+^, Er^3+^ materials.

### Room-Temperature Luminescence

**Figure 1:**
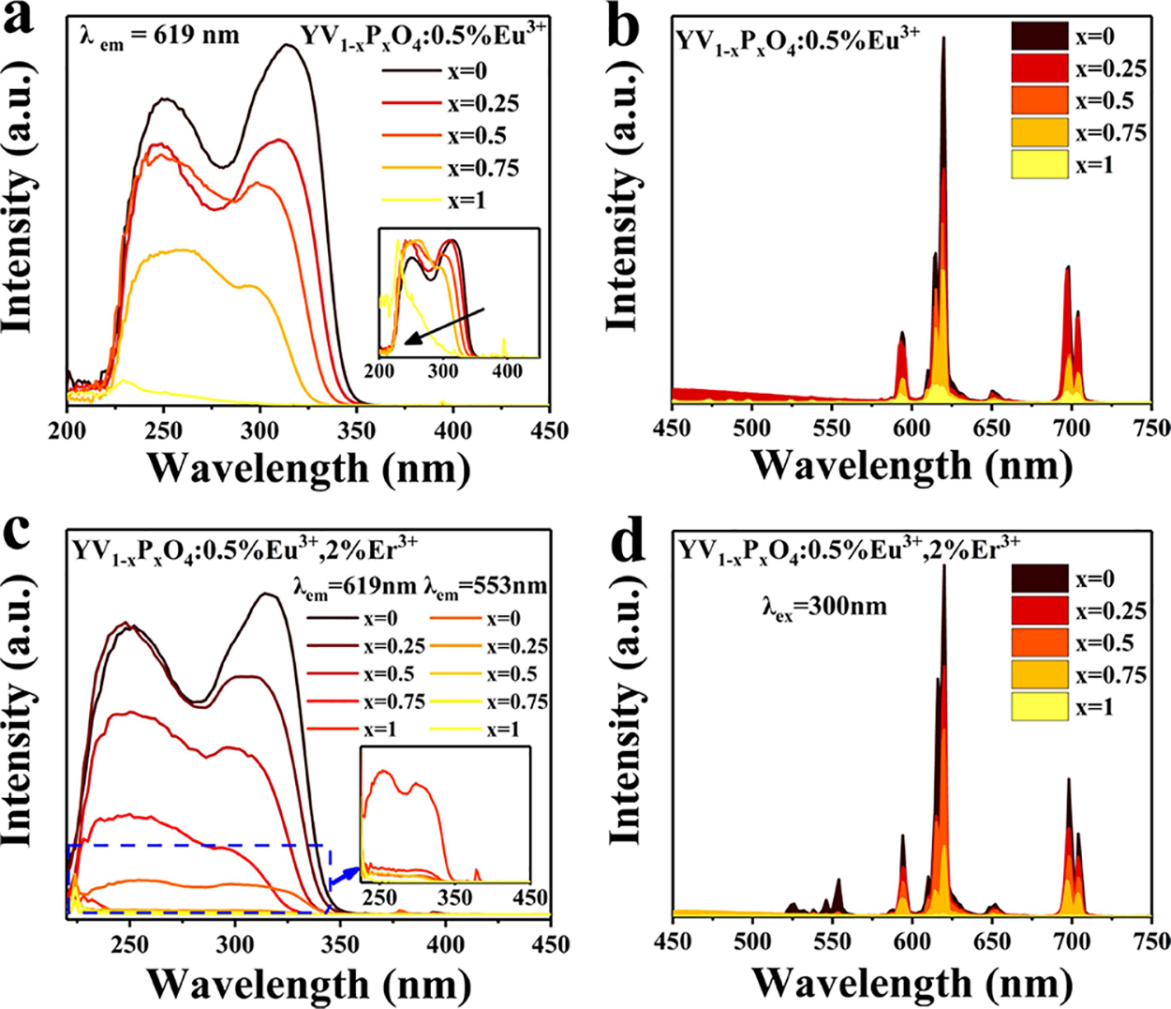
(a) Photoluminescence excitation (PLE) and (b) photoluminescence emission (PL) spectra of YV_1–*x*_P_*x*_O_4_:0.5%Eu^3+^ (*x* = 0, 0.25, 0.5, 0.75 and 1) microcrystals (the inset shows the normalized excitation spectra for Eu^3+^ 619 nm emission). (c) Excitation and (d) emission spectra of YV_1–*x*_P_*x*_O_4_:0.5%Eu^3+^, 2%Er^3+^ (*x* = 0, 0.25, 0.5, 0.75, and 1) microcrystals at room temperature.

To investigate the optical properties, luminescence spectra were measured for the as-prepared singly doped Eu^3+^ microcrystals at room temperature. The excitation spectrum monitored for 619 nm emission in Figure 1a shows a strong and broad near-ultraviolet absorption band originating from the vanadate charge transfer (CT) transition.^41,42^ This is a well-known sensitizer for Ln^3+^ emission. A shift of the CT edge toward higher energies was observed with increasing P content in the solid solution. This blue shift has been attributed to a weakening of the interaction between vanadate groups by dilution with phosphate groups.^43^ As shown in Figure 1b, under excitation at 310 nm, the characteristic Eu^3+^ emission spectrum is observed, showing the orange–red Eu^3+5^D_0_-^7^F_1–4_ transitions.^44^ The ^5^D_0_-^7^F_2_/^5^D_0_-^7^F_1_ intensity ratio increases for a higher VO_4_ content (Figure S3). This has been explained by the hypersensitive nature of the ^5^D_0_-^7^F_2_ transition, which is strongly enhanced if the coordinating ligands are more polarizable, and also by a shift to lower energies of the CT band.^45^ Furthermore, the emission intensity of Eu^3+^ decreases with an increase in the P content. This is explained by less absorbing VO_4_ groups and less efficient energy transfer from VO_4_ to Eu because energy transfer between VO_4_ groups is hampered.^46,47^

The luminescence properties of codoped YV_1–*x*_P_*x*_O_4_:0.5%Eu^3+^, 2%Er^3+^ (*x* = 0, 0.25, 0.5, 0.75, and 1) microcrystals were also analyzed at room temperature. The excitation spectra monitored at 619 nm for Eu^3+^ and 553 nm for Er^3+^, respectively, are shown in Figure 1c. The spectrum exhibits a strong and broad absorption band in UV (vanadate CT), together with weak and narrow absorption lines at 396 nm (Eu^3+^ absorption). Note that the intensity of the CT bands monitored for Eu^3+^ emission is much higher than that of Er^3+^, implying that the energy transfer efficiency from vanadate CT to Eu^3+^ is higher than that to Er^3+^. This is in agreement with a much lower Er^3+^ emission intensity under CT excitation, even though the Er^3+^ concentration is four times higher than that of Eu^3+^ (Figures 1d and S2). These observations are consistent with earlier reports, where a much smaller critical distance for energy transfer from vanadate to Er^3+^ than to Eu^3+^ was calculated.^48^ The origin of the more efficient VO_4_^3–^-to-Eu^3+^ energy transfer is not well understood. For both ions, there is a good spectral overlap of the vanadate emission with Ln^3+^ absorption lines, and for dipole–dipole energy transfer, no large difference in transfer rate is expected. Possibly, wave function overlap-mediated energy transfer (exchange interaction) and the fact that the vanadate and Eu^3+^ CT states are similar in energy, both involving a hole delocalized over the O^2–^ ligands, play a role in the higher energy transfer efficiency for VO_4_^3–^-to-Eu^3+^.

### Lifetime-Based Luminescence Thermometry YV_1–*x*_P_*x*_O_4_:0.5%Eu^3+^

**Figure 2:**
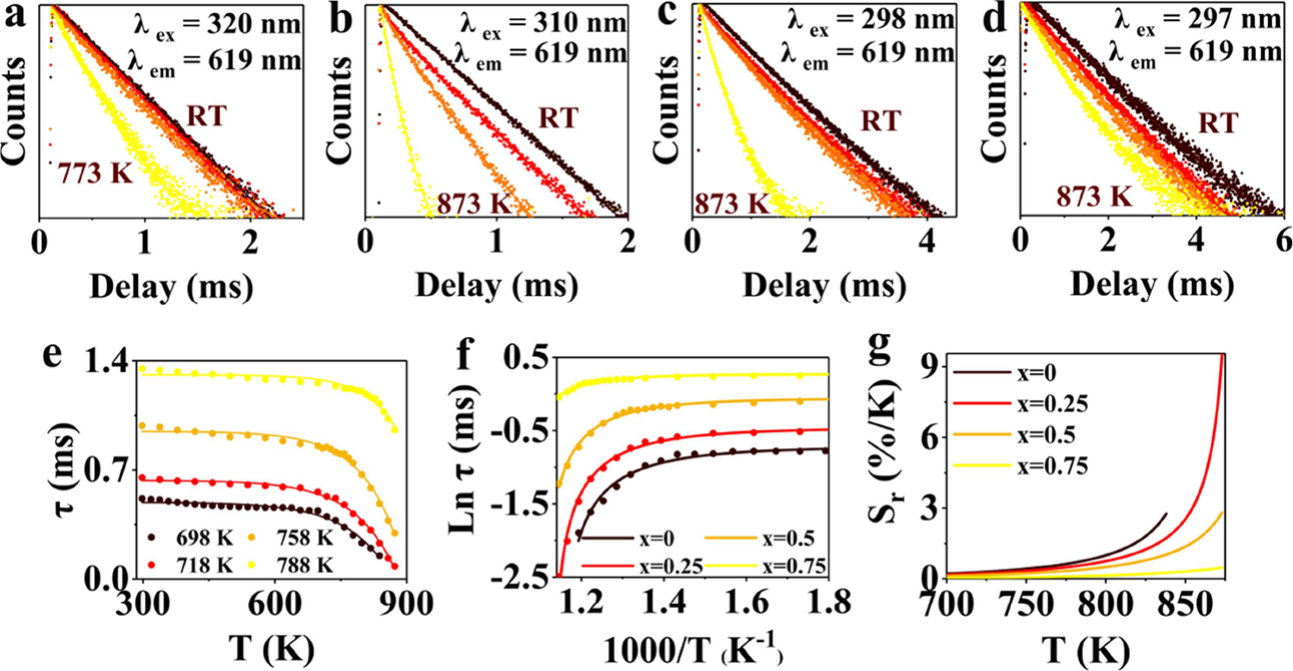
Temperature-dependent luminescence decay curves of the Eu^3+^ emission for single-doped YV_1–*x*_P_*x*_O_4_:0.5%Eu^3+^ microcrystals for (a) *x* = 0, (b) *x* = 0.25, (c) *x* = 0.5, and (d) *x* = 0.75. Note the change in the scale for the time axis. (e) Average lifetime (τ_ave_) of Eu^3+^ emission in YV_1–*x*_P_*x*_O_4_:0.5%Eu^3+^ (*x* = 0, 0.25, 0.5, and 0.75) microcrystals as a function of temperature. (f) Plot of the logarithm of the lifetime (ln τ) versus the inverse of the temperature (1/*T*). (g) Plot of *S*_r_ for the temperature-dependent luminescence lifetime YV_1–*x*_P_*x*_O_4_:0.5%Eu^3+^ (*x* = 0, 0.25, 0.5, and 0.75) microcrystals versus *T*.

In order to investigate the effect of the phosphate/vanadate ratio in the solid solution on the thermal quenching temperature of Eu^3+^, temperature-dependent luminescence lifetime measurements were performed from RT to 873 K (Figure 2a–d). For Eu^3+^ emission in pure yttrium vanadate microcrystals, the decay curves are close to single exponential and therefore can be well fitted by a single-exponential function, while the decay curves for the mixed vanadate–phosphate microcrystals were fitted by a double-exponential function. The nonexponential character in the mixed crystals can be understood based on the variation in local vanadate/phosphate coordination for different Eu^3+^ ions, which affects the decay time. The average lifetimes were calculated using the formula:^49^. At 298 K, the lifetimes for Eu^3+5^D_0_ emission YV_1–*x*_P_*x*_O_4_ (with *x* varying from 0 to 0.75) are 0.52, 0.65, 0.99, and 1.35 ms (Table S1, Figure S4), respectively, consistent with the literature.^34,50^ The hypersensitive ^5^D_0_-^7^F_2_ emission becomes stronger for an increase in concentration of the more polarizable VO_4_ groups vs PO_4_.^36^ This causes the decay time to drop with VO_4_ content, in line with earlier results and theory.

To obtain insight into the thermal quenching of the Eu^3+^ emission, luminescence lifetimes were measured as a function of temperature. In Figure 2a–d, the luminescence decay curves are shown between RT and 873 K for YV_1–*x*_P_*x*_O_4_:0.5%Eu^3+^ (*x* = 0, 0.25, 0.5, and 0.75). The luminescence lifetimes were determined using the equation for τ_ave_ (*vide supra*) and are depicted in Figure 2e. As can be clearly observed from Figure 2e, with an increase in P solid solution content, the lifetime of Eu^3+^ becomes longer, and thermal quenching (*T*_Q_) shifts to higher temperatures. As far as we are aware, the thermal quenching of Eu^3+^ emission in YVO_4_ has not been reported before. The onset temperatures (*T*_onset_) of the thermal quenching for different V/P contents are ∼700, 720, 760, and 790 K upon varying *x* from 0 to 0.75. Also, for increasing phosphate/vanadate ratios, the τ_50_ values (temperature at which the lifetime has dropped to 50% of the low-temperature plateau value) increase and are approximately 788, 798, and 838 K, and exceed 873 K, respectively. This shift to higher *T*_Q_ values for increasing phosphate content can be explained by quenching via the CT state. Upon increasing the PO_4_ content, the CT state shifts to higher energies, resulting in a larger activation energy for thermal quenching as the CT band shifts to higher energies.^42,51,52^

Based on the strong temperature dependence of the Eu^3+^ emission lifetime, precise temperature sensing over a wide range (RT–873 K) can be achieved. By tuning the thermal quenching temperature of Eu^3+^ through composition (P/V ratio) in the mixed vanadate–phosphate yttrium crystal, the temperature range can be optimized for the temperature regime of interest. The temperature dependence of the emission lifetimes τ_ave_ is shown in Figure 2e. The Arrhenius-type behavior observed indicates that quenching is a thermally activated process and can be well fitted by the exponential function:
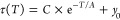
1where *T* is the temperature, *C* is the pre-exponential factor, *A* is the fitting parameter reflecting the activation energy, and *y*_0_ is the constant. The fit parameters for different *x*-values are detailed in Table S2. As a result, luminescence thermometry calibration curves based on temperature-dependent lifetimes were obtained. In order to investigate the sensitivity of temperature sensing for this system, all the relative sensitivities (*S*_r_) were calculated using the following equation:^53^
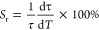
2

High values of *S*_r_ allow for more precise temperature sensing, although other factors, such as signal strength, also help to improve accuracy in temperature sensing. In Figure 2g, the temperature regions, where *S*_r_ is greater than 0.5%/K, are selected as an indication for reliable temperature sensing. For YV_1–*x*_P_*x*_O_4_:0.5%Eu^3+^ microcrystals, within the temperature range 560–873 K, *S*_r_ consistently exceeds 0.5%/K, indicating that the temperature sensing system is more suitable for high-temperature environments. Additionally, as the temperature increases, *S*_r_ rises rapidly beyond an onset temperature (*T*_sens_), e.g., for the YVO_4_:0.5%Eu^3+^ thermometer, the sensitivity increases strongly above 700 K. For *x* = 0.25, *T*_sens_ is approximately 720 K; for *x* = 0.5, *T*_sens_ is around 760 K; and for *x* = 0.75, it is about 830 K. These values coincide with the temperature at which the thermal quenching of Eu^3+^ emission starts. Moreover, with an increase in PO_4_ content, the region of sharp increase in *S*_r_ shifts toward higher temperatures and shows that a suitable phosphate/vanadate ratio can be selected to achieve the optimum sensitivity for a specific temperature range. For microcrystals with *x* = 0.75, the region of steep increase in *S*_r_ is not fully measured, and the optimal temperature sensing region will exceed 873 K. The maximum relative sensitivities (*S*_rmax_) calculated from the available data are 2.8, 9.6, 2.8, and 0.5%/K at 873 K for *x* = 0, 0.25, 0.50, and 0.75, respectively. To test the stability and reproducibility of the performance as a luminescent thermometer, cycling experiments were done for YV_0.5_P_0.5_O_4_:0.5%Eu^3+^,2%Er^3+^ microcrystals over five consecutive heating and cooling cycles from 298 to 873 K at 100 K intervals. Excellent reproducibility is observed and demonstrates that the Y(V,P)O_4_:Er^3+^, Eu^3+^ temperature probes have good stability.

#### Multi-LIR-Based Luminescence Thermometry

Incorporation of lanthanide ions and combinations of lanthanide ions into mixed vanadate–phosphate (nano)crystals is explored to measure the temperature-dependent emission intensity ratio of different emission lines to accurately determine the temperature over a wide temperature range. The charge transfer absorption shifts to lower energy for increasing vanadate content.^30,54,55^ This is used for tuning the quenching temperature for dopant emissions and can help to optimize *S*_r_ for a specific temperature range, not only for temperature-dependent lifetimes but also affecting the temperature dependence of luminescence intensity ratios (LIRs).

To investigate the feasibility of this approach, variable-temperature emission spectra were recorded between RT and 873 K with intervals of 20 K for YV_1–*x*_P_*x*_O_4_:0.5%Eu^3+^, 2%Er^3+^ with *x* = 0, 0.25, 0.5, and 0.75. By selecting different temperature-dependent LIRs and temperature ranges, this system can simultaneously utilize two intensity ratio temperature sensing parameters for temperature detection. One parameter is the well-known temperature dependence of the LIR of the Er^3+2^H_11/2_-^4^S_3/2_ thermally coupled energy levels, which is affected by the thermal equilibrium of ^2^H_11/2_ and ^4^S_3/2_ of Er^3+^ (Boltzmann temperature probe). The other relies on the strong temperature dependence of the Er^3+^/Eu^3+^ LIR, which is influenced by the difference in thermal quenching of the Er^3+^ and Eu^3+^ emission. Note that as *x* increases from 0 to 0.75, the thermal quenching temperature of Eu^3+^ increases, and the temperature region of highest sensitivity shifts to higher temperatures.

**Figure 3:**
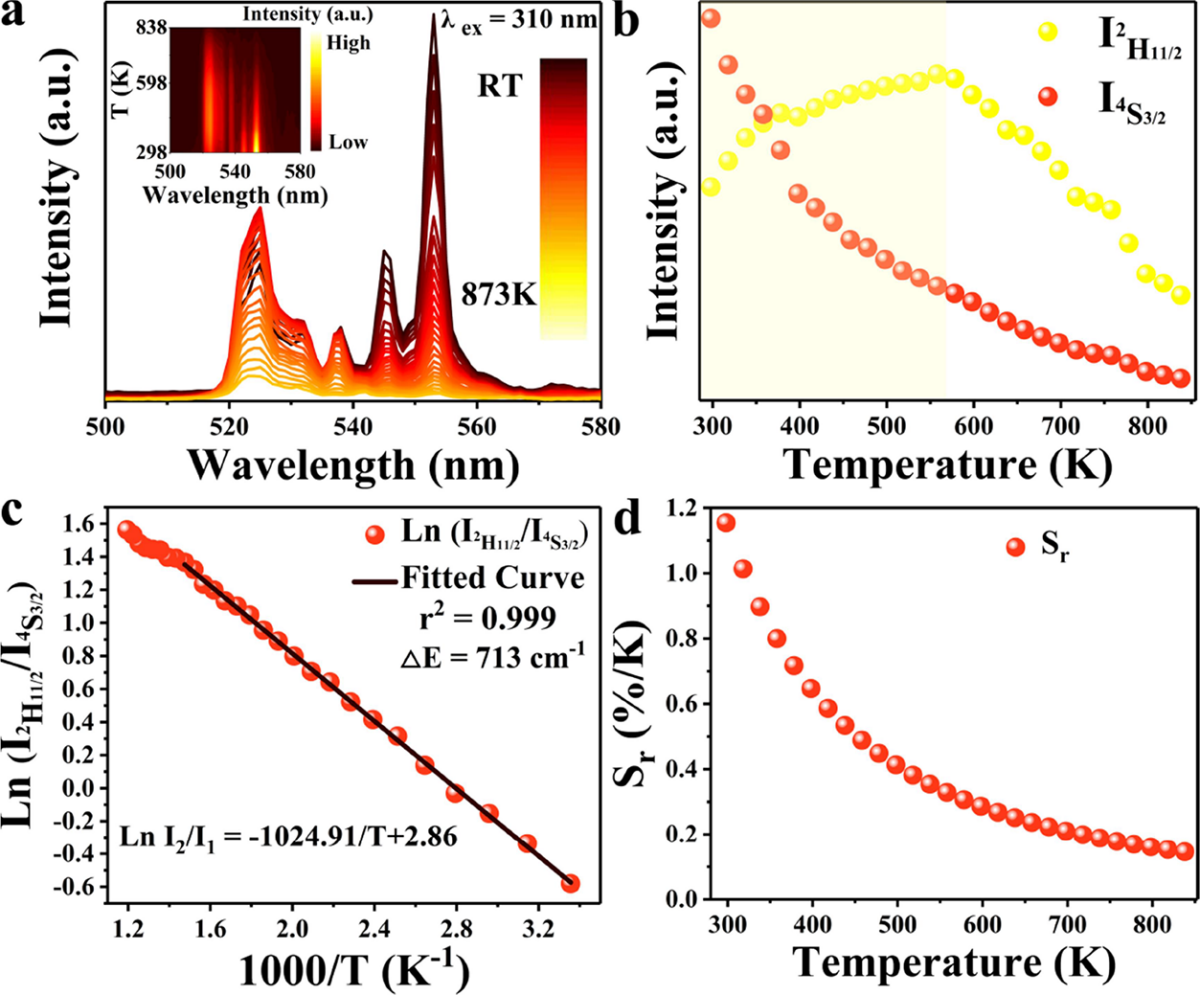
(a) Temperature-dependent emission spectra of YVO_4_:0.5%Eu^3+^ and 2%Er^3+^ for Er^3+2^H_11/2_-^4^S_3/2_ emission under 310 nm excitation. (b) Integrated intensities of ^2^H_11/2_-^4^I_15/2_ and ^4^S_3/2_-^4^I_15/2_ emissions with the temperature. (c) Ln (*I*_^2^H_11/2__/(*I*_^4^S_3/2__) as a function of 1/*T*. (d) *S*_r_ as a function of temperature for temperature sensing based on the ^2^H_11/2_-^4^S_3/2_ LIR for YVO_4_:0.5%Eu^3+^, 2%Er^3+^ microcrystals.

The well-known Er^3+2^H_11/2_-^4^S_3/2_ Boltzmann thermometry is first analyzed using YVO_4_:Er^3+^, Eu^3+^ as an example. The emission spectra at elevated temperatures, monitored for 310 nm excitation in the vanadate charge transfer band, were measured over a temperature range of 298–873 K. In order to observe the variation of Er^3+^ emission intensity with temperature more clearly, Figure 3a shows an enlarged view of the Er^3+^ emission in the range of 500–580 nm. As the temperature increases, the emission intensity of the Er^3+2^H_11/2_-^4^I_15/2_ transition (*I*_2_) initially increases and then decreases, while the emission intensity of the ^4^S_3/2_-^4^I_15/2_ transition (*I*_1_) continuously decreases (Figure 3b). The increase in the ^2^H_11/2_ emission is due to the well-known Boltzmann equilibrium between the ^4^S_3/2_ and ^2^H_11/2_ levels. The decrease at even higher temperatures is caused by the effects of temperature quenching.^56^ In Figure 3c, the logarithmic plot of the Ln LIR (^2^H_11/2_/^4^S_3/2_) vs 1/*T* shows the expected linear dependence calibration line. The temperature dependence analysis of the LIR is performed using the formula:^56^
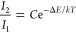
3

Within the temperature range of 300–600 K, a good linear relationship (*r*^2^ = 0.999) is observed. Here, *I*_2_ represents the integrated emission intensity of the ^2^H_11/2_-^4^I_15/2_ transition within the wavelength range of 516–540 nm, and *I*_1_ represents the integrated emission intensity of the ^4^S_3/2_-^4^I_15/2_ transition within the wavelength range of 540–568 nm. The fitted calibration equation obtained is ln LIR = −1025/*T* + 2.86. From the fitted parameters, the slope ΔE/k is determined to be 1025, corresponding to a Δ*E* of 713 cm^–1^ for the ^2^H_11/2_-^4^S_3/2_ energy difference, consistent with previous reports.^28,31,56^ There is a deviation in the high-temperature region, which may be due to higher excited states becoming populated. Based on the temperature calibration line, *S*_r_ for temperature sensing performance was determined (Figure 3d). Within the temperature range of 298–873 K, *S*_r_ decreases with increasing temperature and exhibits a value for *S*_rmax_ of 1.2%/K around room temperature. This behavior is typical for the ^2^H_11/2_/^4^S_3/2_ LIR of Er^3+^.^57^ The *S*_*r*_ of this type of thermometer depends on Δ*E* and decreases with temperature as *S*_r_ = Δ*E*/*k*_B_*T*^2^, where *k*_B_ is the Boltzmann constant. *S*_r_ is above 0.5%/K only up to ∼450 K for the ^2^H_11/2_/^4^S_3/2_ LIR.

**Figure 4:**
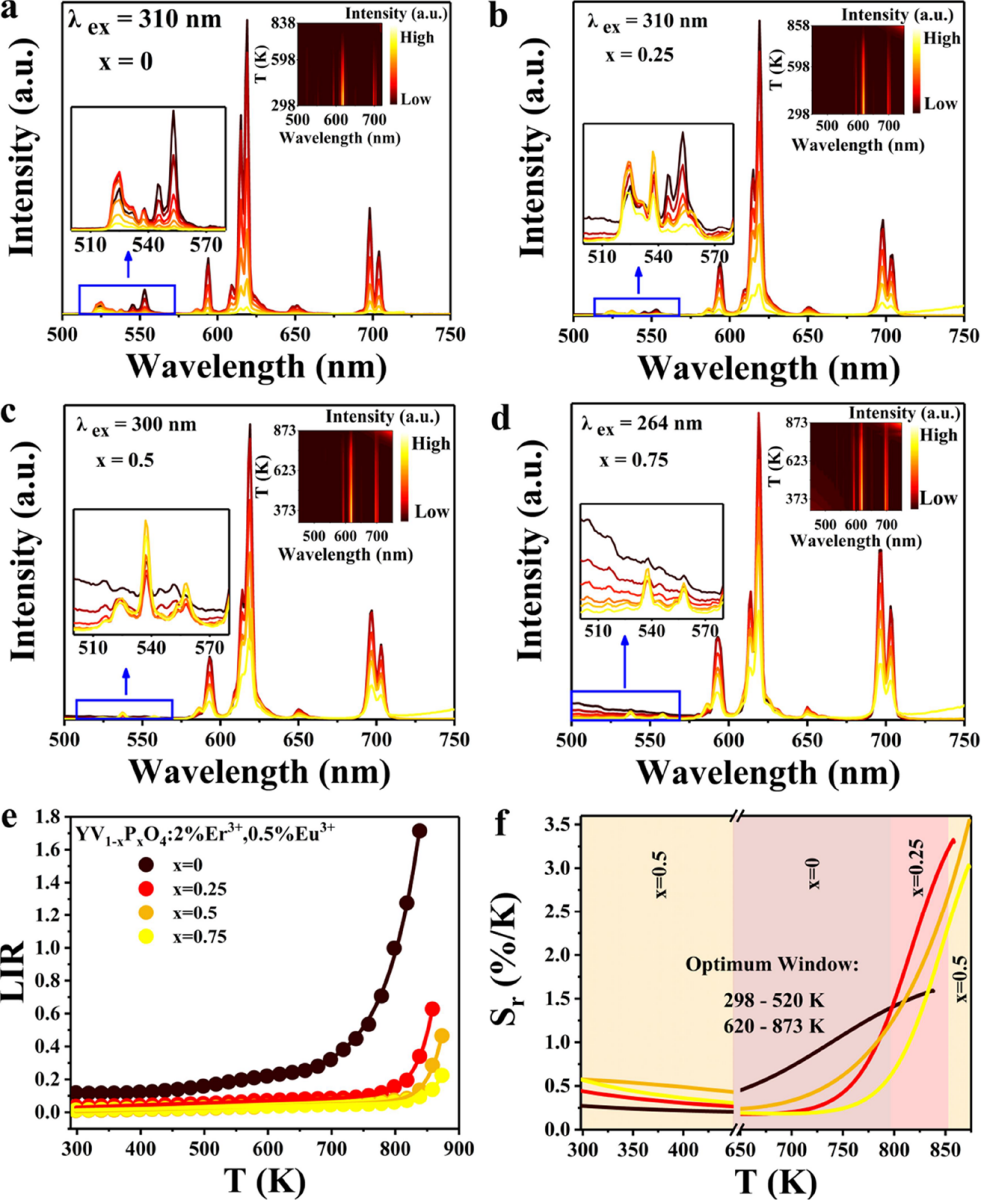
Variable temperature emission spectra of YV_1–*x*_P_*x*_O_4_:0.5%Eu^3+^, 2%Er^3+^ microcrystals for (a) *x* = 0, (b) *x* = 0.25, (c) *x* = 0.5, and (d) *x* = 0.75. (e) Total Er^3+^/Eu^3+^ LIR-based temperature sensing of YV_1–*x*_P_*x*_O_4_:0.5%Eu^3+^, 2%Er^3+^ (*x* = 0, 0.25, 0.5, and 0.75) microcrystals. (f) Optimization diagram of *S*_r_ as a function of *T*. Excitation wavelengths are indicated in the figures.

Further research was conducted on the temperature-dependent LIR based on the ratio of the Er^3+^/Eu^3+^ emission. The variable-temperature emission spectra of YV_1–*x*_P_*x*_O_4_:0.5%Eu^3+^,2%Er^3+^ (*x* = 0, 0.25, 0.5, and 0.75) microcrystals were monitored at 20 K intervals from RT to 873 K. The spectra in Figure 4a–d show weaker Er^3+^ and stronger Eu^3+^ emission, which reflects less efficient sensitization of Er^3+^. The low-intensity emission of Er^3+^ has an overlap with the vanadate host emission and requires background subtraction. After background subtraction, the ratio of *I*_Er_^3+^/*I*_Eu_^3+^ (where *I*_Eu_^3+^ represents the integrated emission intensity in the range of 580–640 nm and *I*_Er_^3+^ represents the integrated emission intensity of ^2^H_11/2_ + ^4^S_3/2_) with temperature is shown in Figure 4e. The difference in the thermal behavior of Er^3+^ and Eu^3+^ emissions allows for temperature sensing based on the Er^3+^/Eu^3+^ LIR. Thermal quenching of the Eu^3+^ emission causes an increase in the LIR value, and the onset of quenching shifts to higher *T* for increasing phosphate content.

The experimentally observed relationship between LIR (*I*_Er_^3+^/*I*_Eu_^3+^) and T (Figures S5 and S6) can be well fitted by an empirical formula, including the combination of an exponential and linear function within the tested temperature range (298–873 K). The resulting temperature calibration curve is given by the following equation:
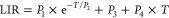
4where *P*_1_ to *P*_4_ are fitting parameters. As *x* varies from 0 to 0.75, the aforementioned equation can be used to obtain temperature calibration lines for all four YV_1–*x*_P_*x*_O_4_:0.5%Eu^3+^,2%Er^3+^ systems. The fitting parameters are detailed in Table S3.

To appreciate the performance of the YV_1–*x*_P_*x*_O_4_:0.5%Eu^3+^,2%Er^3+^ thermometers and to gain a full understanding of the optimum temperature sensing range for different P solid solvents, the *S*_r_ values of four materials with x ranging from 0 to 0.75 were calculated. Figure 4f clearly shows an increasing trend of *S*_r_ with the increase in temperature, which rapidly increases beyond 650 K, indicating that the extension to higher temperature ranges has been effectively achieved. Furthermore, *S*_rmax_ is 3.6%/K at 873 K for *x* = 0.5. Different temperature range requirements can be realized for optimal V/P ratios. For 298–520 K, *x* is chosen as 0.5; for 620–790 K, 0% P; and for 790–850 K, *x* equals 0.25, which has the optimal *S*_r_, and beyond 850 K, 50% P content can be used. In this way, by changing the composition of the matrix and selecting different values of *x*, the optimization of the temperature sensing performance has been successfully realized.

#### Multi-LIR Temperature Sensing in Nanocrystalline YV_1–*x*_P*_*x*_*O_4_:Eu^3+^, Er^3+^

Luminescence thermometry relying on luminescent nanocrystals can offer high spatial resolution (diffraction limit) for temperature mapping,^58−60^ and the smaller size allows for faster response to temperature changes, enabling more precise temperature measurements. Additionally, they can be embedded into microdevices or nanostructures, enabling real-time monitoring and control of local temperatures, as demonstrated in extensive recent work on nanocrystalline Ln-doped luminescent temperature sensors.^10,20,61,62^ Y(P,V)O_4_ is a well-known material for making small (down to ∼4 nm) nanocrystals.^32^

**Figure 5:**
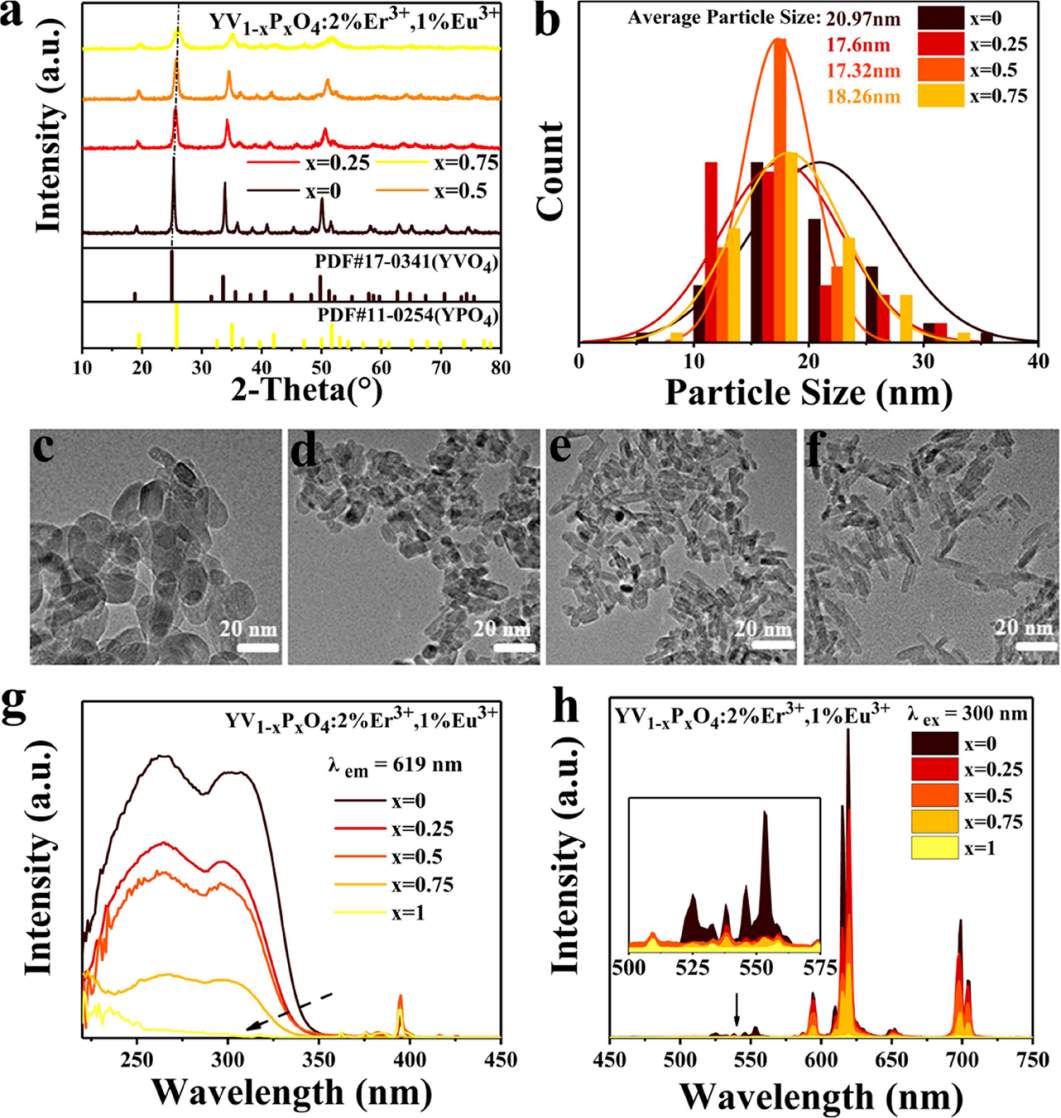
(a) XRD patterns showing a single phase of YV_1–*x*_P_*x*_O_4_:2%Er^3+^,1%Eu^3+^ (*x* = 0, 0.25, 0.5, and 0.75) nanoparticles. (b) Particle size distribution of YV_1–*x*_P_*x*_O_4_:2%Er^3+^, 1%Eu^3+^ (*x* = 0, 0.25, 0.5, and 0.75) nanoparticles determined from TEM images of YV_1–*x*_P_*x*_O_4_:2%Er^3+^, 1%Eu^3+^ nanoparticles for P solid solution amounts of (c) *x* = 0, (d) *x* = 0.25, (e) *x* = 0.5, and (f) *x* = 0.75. (g) Excitation spectra of YV_1–*x*_P_*x*_O_4_:2%Er^3+^, 1%Eu^3+^ (*x* = 0, 0.25, 0.5, 0.75, and 1) nanocrystals monitoring Eu^3+^ 619 nm emission. (h) Emission spectra of YV_1–*x*_P_*x*_O_4_:2%Er^3+^, 1%Eu^3+^ (*x* = 0, 0.25, 0.5, 0.75, and 1) nanocrystals under 300 nm excitation (the inset shows the emission spectrum of Er^3+^ in the range of 500–575 nm).

High-quality nanocrystals of YV_1–*x*_P_*x*_O_4_:2%Er^3+^,1%Eu^3+^ (*x* = 0, 0.25, 0.5, and 0.75) were obtained through the hydrothermal method (Figure 5a).^63,64^ The morphology and average particle size of the nanocrystals were analyzed using TEM, as shown in Figure 5b–f. With the increase in P solid solution content, the nanocrystals transitioned from a block-like shape to a rod-like structure, with an average particle size from 17 to 21 nm. The dispersibility of all nanocrystals remains unchanged. The variation in shape and size may stem from changes in composition and synthesis conditions.

Room-temperature excitation and emission spectra were recorded for comparison with those of the microcrystalline materials. Due to the lower luminescence intensity of Eu^3+^ doping at 0.5%, the doping amount of Eu^3+^ was adjusted to 1%. As shown in Figure 5g, the luminescence spectra are similar to those recorded for the microcrystalline material. The CT onset shifts to higher energies as the amount of P in the solid solution increases.^65^ The excitation peak intensity monitored at Eu^3+^ 619 nm emission is much stronger than the excitation spectrum monitored at Er^3+^ 553 nm, again demonstrating that the energy transfer from the vanadate CT to Eu^3+^ is more efficient, which can also be observed in the emission spectra under 300 nm excitation, as shown in Figure 5h.

**Figure 6:**
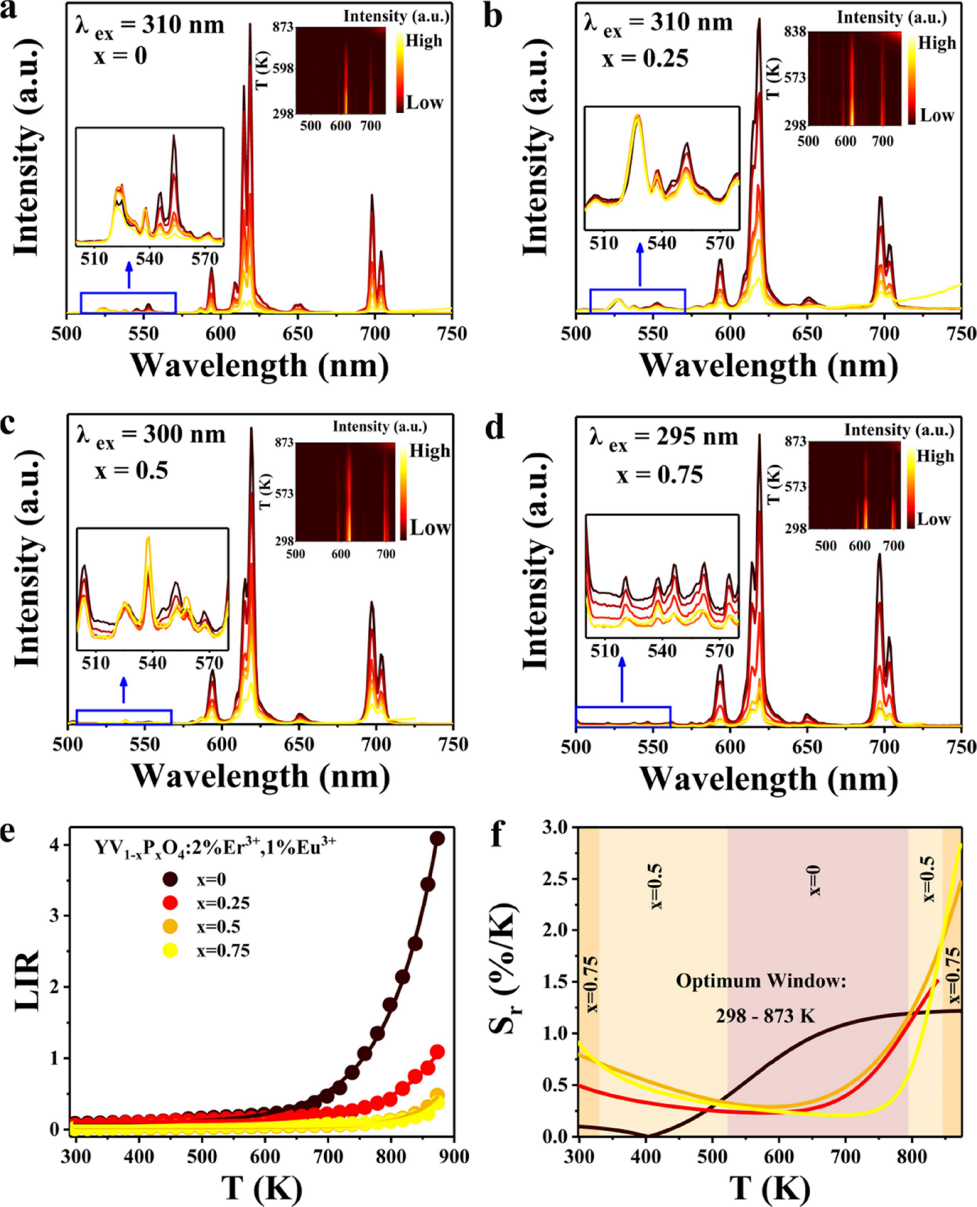
Variable temperature emission spectra of YV_1–*x*_P_*x*_O_4_:2%Er^3+^,1%Eu^3+^ (*x* = 0, 0.25, 0.5, and 0.75) nanocrystals for (a) *x* = 0, (b) *x* = 0.25, (c) *x* = 0.5, and (d) *x* = 0.75. (e) Er^3+^/Eu^3+^ LIR-based temperature sensing of YV_1–*x*_P_*x*_O_4_:1%Eu^3+^,2%Er^3+^(*x* = 0, 0.25, 0.5, and 0.75) nanocrystals. (f) Diagram of *S*_r_ as a function of *T*.

The emission spectra of YV_1–*x*_P_*x*_O_4_:2%Er^3+^,1%Eu^3+^ (*x* = 0, 0.25, 0.5, and 0.75) nanocrystals were monitored from RT to 873 K at intervals of 20 K, as shown in Figure 6a–d. After careful background subtraction, the Er^3+2^H_11/2_-^4^S_3/2_ thermally coupled levels allowed for temperature sensing based on Boltzmann thermal equilibrium (as discussed earlier, see the Supporting Information for details, Figure S7), and a linear calibration curve was obtained. The slope obtained from this linear fit allows for the calculation of Δ*E* for ^4^S_3/2_-^2^H_11/2_ as 713 cm^–1^, consistent with the T-sensing of Er^3+^ in the microcrystalline system.

The higher Eu-concentration and differences in quenching of Er and Eu emission in nanocrystals vs microcrystals can cause differences in the LIR for Eu, Er-codoped NCs compared to microcrystals. The thermal quenching behavior of Eu^3+^ occurs at higher temperatures upon adding more P in the solid solution and allows for temperature sensing using YV_1–*x*_P_*x*_O_4_:2%Er^3+^,1%Eu^3+^ nanocrystals over wide temperature ranges. By combination of these two parameters/models, precise temperature sensing can be achieved. Specifically, as the temperature increases, the change in intensity ratio of Er^3+2^H_11/2_/^4^S_3/2_ emission can be explained by the well-known Boltzmann thermal equilibrium, while the change in emission intensity of Eu^3+^ is influenced by thermal quenching via the CT state. Plotting the Er^3+^/Eu^3+^ LIR versus *T* (Figure 6e), where the integration region of Er^3+^ and Eu^3+^ emission remains consistent with the microcrystalline system, as shown in Figures S8 and S9, the variation of LIR versus *T* can be well fitted by the following empirical function over the entire temperature region:
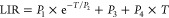
5

This empirical fit is similar to the function used for the microcrystalline system. The fitting parameters are listed in Table S4. From the results, it is evident that Eu^3+^ undergoes quenching at higher temperatures as *x* increases from 0 to 0.75. *S*_r_ was calculated as a function of *T* (Figure 6f). Similar to the microcrystalline system, there is a consistent trend of increasing sensitivity with increasing temperature, and high relative sensitivities are in the high-temperature region (>700 K) with *S*_rmax_ being 2.9%/K for *x* = 0.75 at 873 K. Throughout the temperature region of the RT–873 K, different *x* values can be used to ensure optimal temperature sensing performance.

In summary, the relationship between *S*_r_ and the temperature indicates that the probe becomes more sensitive in the high-temperature region as the P content increases. This enables our YV_1–*x*_P_*x*_O_4_:Er^3+^,Eu^3+^ luminescence (micro/nano)thermometry to achieve optimal temperature sensing over a wide temperature range (RT–873 K). Furthermore, by combining temperature sensing parameters (Boltzmann type + multi-LIR model), more accurate temperature measurement results can be achieved.^66^

## Applications

### Luminescence Thermometry for Chip Temperature Detection

A potential application of luminescent thermometers is monitoring chip temperature for performance optimization, fault prediction, protection against overheating, chip design and optimization, and system security and stability. It is important to ensure normal operation and improve the reliability of the chip. Leveraging the advantages of our new luminescent thermometer in high sensitivity and precise temperature measurement through multiple temperature sensing modalities, we encapsulated a YVO_4_:0.5%Eu^3+^ luminescence microthermometer and a YVO_4_:2%Er^3+^,1%Eu^3+^ luminescence nanothermometer on the surface of the RT9955 chip for temperature detection. The RT9955 chip provides a complete power supply solution for digital still cameras and other hand-held devices. To realize temperature sensing, the micro- or nanocrystallites are thoroughly mixed with the adhesive and coated onto the surface of the chip. After curing, the temperature-dependent luminescence was used for chip temperature detection (see detailed photos in the SI, Figure S10). The chip coated with the T-sensor is placed in a temperature-controlled device to monitor temperature changes. This temperature-controlled device can be connected to a computer to control the temperature (*T*_set_) and obtain real-time temperature (*T*_real_) change curves. It is connected to a spectrometer to detect the spectrum of the T-sensor. When the two points *T*_set_ and *T*_real_ coincide, the emission spectrum is recorded. The recorded spectra, combined with the calibration curves obtained earlier, are used to calculate a temperature (*T*_C–B_ or *T*_C-M_, here, *T*_C–B_ is the chip temperature calculated using the Er^3+2^H_11/2_-^4^S_3/2_ LIR Boltzmann temperature sensing model and *T*_C-M_ is the chip temperature calculated using Er^3+^/Eu^3+^ LIR thermometry). The temperature error (*T*_Error_) is the difference between *T*_real_ and *T*_C–B_ (or *T*_C-M_).

**Figure 7:**
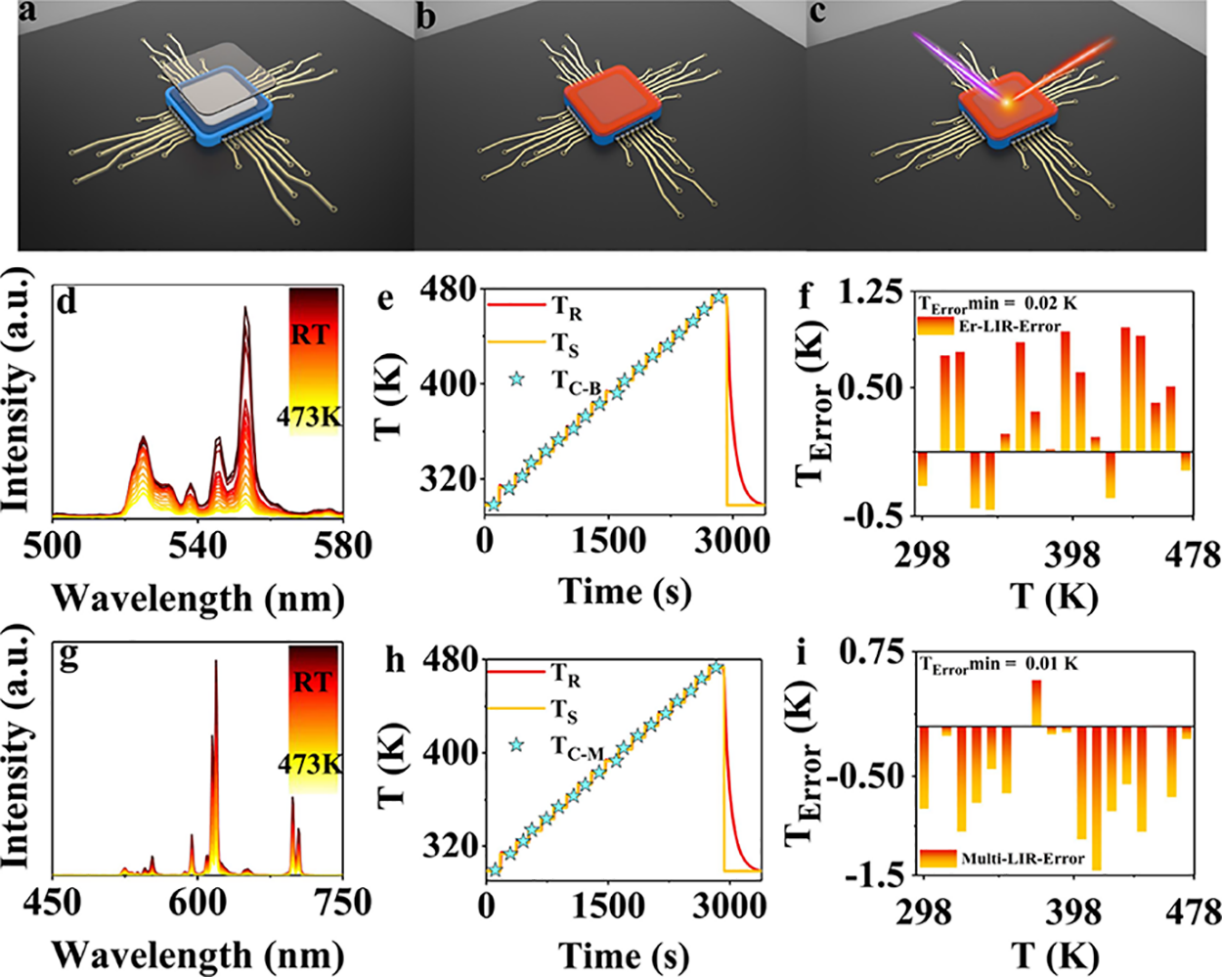
(a–c) Schematic diagrams of the luminescent thermometer for chip temperature probing. (d) Temperature-dependent emission spectra of the chip after packaging with the YVO_4_:2%Er^3+^,1%Eu^3+^ luminescence nanothermometer excited at 310 nm, from RT to 473 K at 10 K intervals. (e) Real-time monitored real temperature (*T*_R_) curve, temperature set (*T*_S_) curve of the variable temperature device, and the chip temperature calculated based on Er^3+2^H_11/2_-^4^S_3/2_ LIR Boltzmann thermometry (*T*_C–B_). (f) *T*_Error_ between *T*_C–B_ and *T*_R_ at different temperatures. (g) Temperature-dependent emission spectra of the chip after packaging with the YVO_4_:2%Er^3+^,1%Eu^3+^ luminescence nanothermometer excited at 310 nm, from RT to 473 K at 10 K intervals. (h) *T*_R_ curve, *T*_S_ curve, and chip temperature calculated based on Er^3+^/Eu^3+^ LIR thermometry (*T*_C-M_). (i) *T*_Error_ between *T*_C-M_ and *T*_R_ at different temperatures.

Figure 7a–c displays schematic diagrams of luminescent thermometers used for chip temperature detection. Under UV illumination, the encapsulated chip exhibits bright orange–red light, which facilitates precise sensing of the chip temperature. The encapsulated chip was placed in a variable temperature device, and the required temperature-dependent emission spectra were monitored under excitation at 310 nm. After heating from RT to 473 K and then cooling back to RT, the luminescent thermometers encapsulated on the chip surface showed no significant changes, indicating the stability of this approach (Figure S10). Note that in this configuration, the chip acts as a sample holder in the heater, and we do not monitor temperature changes under chip operation conditions. Instead, experiments give a good indication of the temperature accuracy and systematic errors using the T-sensors applied to the chip surface.

The performance of real-time chip temperature detection based on Er^3+^ LIR Boltzmann thermometry was studied. The emission spectra were recorded under 310 nm excitation at intervals of 10K between RT and 473 K (Figure 7d). Using the calibration line indicated by eq 3 in the previous section, the chip temperature (*T*_C–B_) at each detection point was acquired. Figure 7e shows good agreement between *T*_C–B_ and the actual temperature *T*_real_ curve, as well as the set temperature *T*_set_ curve. Similarly, by comparing *T*_C–B_ with *T*_real_, the *T*_Error_ at each temperature was obtained, as shown in Figure 7f. The *T*_Error_ obtained based on the Er^3+^ Boltzmann thermometry varies between 0 and 1 K, which indicates a better than 1 K accuracy for chip temperature detection. These temperature accuracies are in agreement with an *S*_r_ around 0.5% K^–1^ and count rates of 10^5^ photons. Based on Poisson statistics for the photon count rate, one can expect a temperature uncertainty of 0.5 K for these rates.^23,67^ Longer integration times may reduce the temperature uncertainty at the expense of longer measuring times.

Also, the performance of chip temperature detection based on the Er^3+^/Eu^3+^ LIR temperature measurement method was investigated. Following the same procedure as described above, emission spectra were recorded at 310 nm excitation at intervals of 10 K, with the temperature detection range from RT to 473 K (Figure 7g). Using the calibration equation shown in the previous section, we obtained the chip temperature (*T*_C-M_). Figure 7h demonstrates a good match between *T*_C-M_ and the *T*_real_ curve, as well as the *T*_set_ curve. Similarly, the *T*_Error_ at each temperature was obtained, as shown in Figure 7i. The *T*_Error_ based on the Er^3+^/Eu^3+^ multi-LIR thermometry varied between 0 and 1.5 K, again demonstrating the excellent performance of our new luminescence nanothermometer for chip temperature detection applications.

## Conclusions and Outlook

The present work outlines the performance of mixed yttrium vanadate–phosphate codoped with Er^3+^ and Eu^3+^ for accurate luminescence temperature sensing. This material utilizes the emission of Ln^3+^ sensitized by strong and broad vanadate charge transfer absorption and has a wide and variable optimum temperature range by controlling the thermal quenching temperature of Eu^3+^ emission through phosphate/vanadate substitution. The new temperature probe offers a single material with multiple temperature-dependent luminescence properties, viz. the LIR of ^2^H_11/2_/^4^S_3/2_ emission of Er^3+^, the LIR of the integrated Er^3+^ and Eu^3+^ emission intensities, and the Eu^3+^ emission lifetime. Both micro- and nanocrystalline temperature probes are reported to achieve *S*_r_ from ∼0.5%/K to over 5%/K in a wide temperature range from RT to 873 K. Finally, to demonstrate practical applicability, the luminescent thermometer was applied to in situ chip temperature detection revealing temperature accuracies better than 1 K, which indicates that our novel luminescent thermometer holds great potential for accurate temperature sensing applications.

## Supporting Information Available

Experimental section; XRD patterns of microcrystals; emission spectra of Er^3+^; calculations of temperature-dependent lifetimes; variable temperature emission spectra of microcrystals; variable temperature emission spectra of nanocrystals; plots of Er^3+^/Eu^3+^ LIR vs *T* of microcrystals; Er^3+^ LIR Boltzmann thermometry of nanoparticles; plots of Er^3+^/Eu^3+^ LIR vs *T* for nanoparticles; photos of the test process using T-sensor for chip temperature detection; and SEM images with EDX elemental mapping and results for composition analysis with ICP-OES (PDF)
