# Supplementary material for: Luminescence Thermometry via Multiparameter Sensing in YV1–xPxO4:Eu3+, Er3+
Source: J Am Chem Soc. 2025 Apr 4;147(15):12925–36. doi: 10.1021/jacs.5c02306 (PMC12007000; doi:10.1021/jacs.5c02306)
Supplement: Supplementary file 1 — ja5c02306_si_001.pdf [file ja5c02306_si_001.pdf]

Supporting Information

Luminescence Thermometry via Multi-Parameter Sensing in $YV_{1-x}P_xO_4$:Eu^{3+}, Er^{3+}

*Yixuan Ma[1], Xiaopeng Zhou[1], Jiapeng Wu[1], Zhijie Dong[1], Lizhi Cui[1], Yuhua Wang[*1] and Andries Meijerink[*1, 2]*

1. National and Local Joint Engineering Laboratory for Optical Conversion Materials and Technology of National Development and Reform Commission, School of Materials and Energy, Lanzhou University, Lanzhou, 730000, China.
E-mail: wyh@lzu.edu.cn

2. Condensed Matter and Interfaces, Debye Institute for Nanomaterials Science, Utrecht University, Princetonplein 1, 3584CC Utrecht, The Netherlands
E-mail: a.meijerink@uu.nl

The Supporting Information contains 17 pages, including 12 Figures and 5 Tables.

Experimental Section

Material and Microcrystalline Synthesis: The purity of the raw materials was Y_2O_3 (99.99%), Eu_2O_3 (99.999%), Er_2O_3 (99.99%),V_2O_5(AR) and $(NH_4)_2HPO_4$(AR). All starting materials were used directly without further purification. Eu^{3+}-doped and Eu^{3+},Er^{3+}-co-doped samples were synthesized by standard two-step solid state synthesis techniques[35-37]. Typically, raw materials were weighed according to the stoichiometry. After thorough grinding with mortar and pestle, the mixture was placed in an alumina crucible and preliminarily fired at 750 °C for 3 h under O_2 for 3 hours in a sealed tube furnace. Subsequently, regrinding and a second fire at high temperature of 1150 °C for 6 h were carried out for completing the reaction. Finally, the sintered samples were left

to cool to room temperature in the furnace and taken out and ground into powder for further analysis.

Material and Nanocrystals Preparation: The raw materials and their purity was Y_2O_3 (99.99%), Eu_2O_3 (99.999%), Er_2O_3 (99.99%), NH_4VO_3(AR) and $(NH_4)_2HPO_4$(AR). First all oxides were dissolved in nitric acid. All nitrates were weighed and dissolved in 50 ml of deionized water according to the stoichiometric ratio. Then, they were fully dissolved and mixed well by vigorous stirring for 1h and adjusted the pH value to 7~8. Next, the solution was placed in 100 ml autoclaves and reacted at 200°C for 16 h. After cooling to room temperature, the samples were centrifuged and washed several times with deionized water and alcohol to remove soluble impurities. The obtained samples were dried at 60°C for 12h.

Measurements and characterization: X-ray powder diffraction (XRD) patterns were recorded using a Bruker D2 PHASER X-ray diffractometer using Cu Kα radiation (generator at 30 kV, 15 mA scanning range 2θ from 10−80°). The morphology and elements of $Y(P,V)O_4:Eu^{3+}$, Er^{3+} materials were examined by scanning electron microscopy (SEM, Hitachi S-3400) and energy-dispersive spectroscopy (EDS). TEM images were obtained using a transmission electron microscope (Tecnai G2 F30, FEI, USA). The photoluminescence emission (PL) and excitation (PLE) spectra were recorded using a Fluorolog-3 spectrofluorometer equipped with a 450 W Xenon lamp excitation source. The variable temperature spectra were measured by an Edinburgh Instruments FLS1000 steady-state/transient fluorescence spectrometer in combination with a heating stage (HCP621G-CUV1). The elemental contents of $Y(P,V)O_4:Eu^{3+}$, Er^{3+} materials were determined on an inductively coupled plasma-optical emission spectrometer (ICP-OES, Agilent720ES).

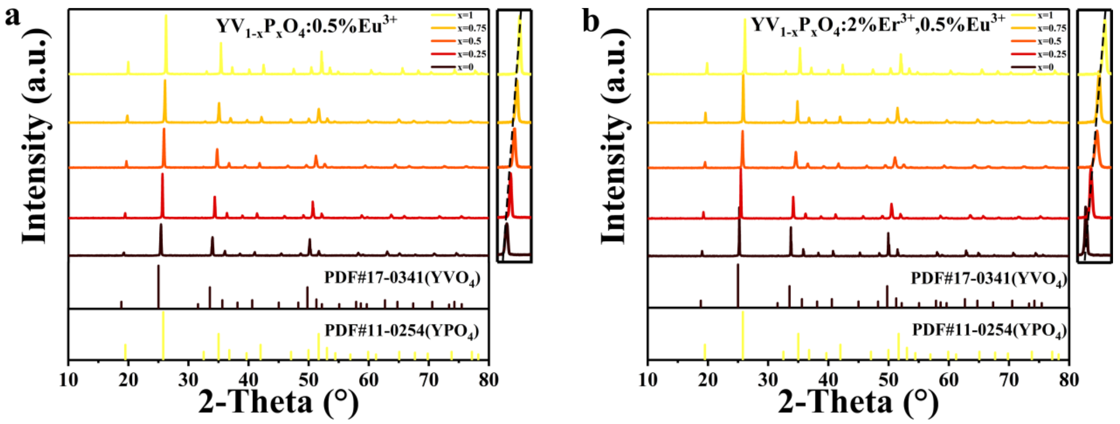

Figure S1. (a) XRD patterns of $YV_{1-x}P_xO_4$:0.5%Eu^{3+} microcrystals. (b) XRD patterns of $YV_{1-x}P_xO_4$:0.5%Eu^{3+}, 2%Er^{3+} (x = 0,0.25,0.5,0.75 and 1) microcrystals. The shift of the strongest diffraction peak around 26° to larger angles for higher PO_4^{3-}content is shown on the right hand side of the figures.

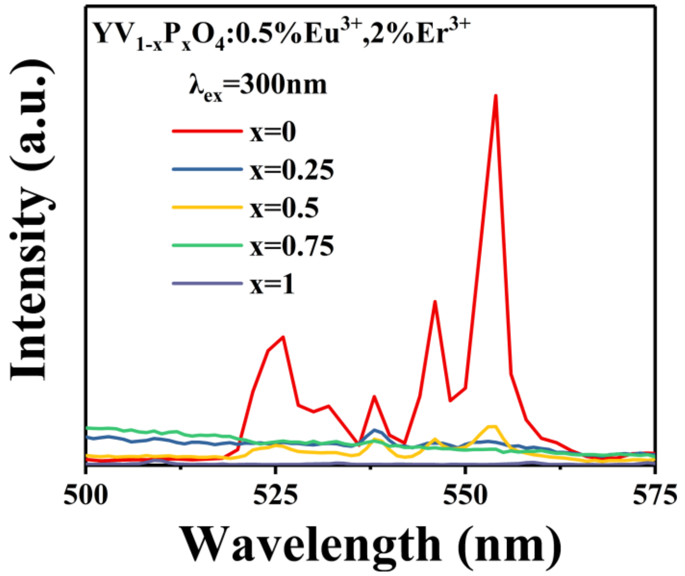

Figure S2. Emission spectrum of Er^{3+} in the range of 500-575nm under 300 nm excitation for $YV_{1-x}P_xO_4$:0.5%Eu^{3+}, 2%Er^{3+} (x = 0, 0.25, 0.5, 0.75 and 1) microcrystals.

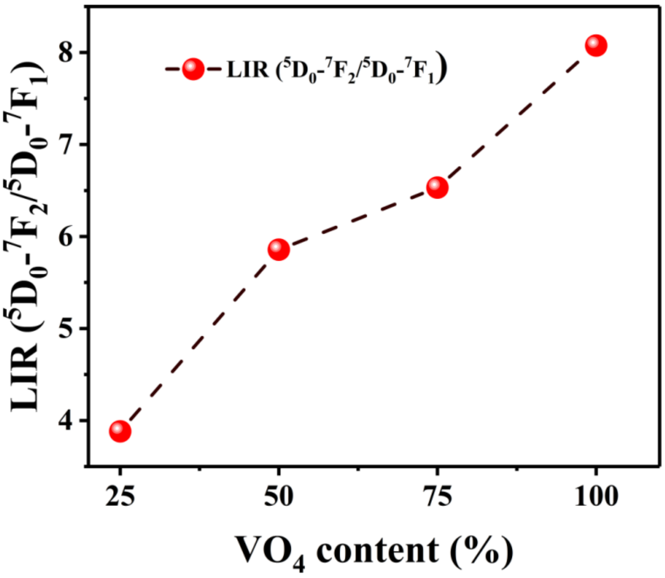

Figure S3. Plot of LIR($^5D_0$-$^7F_2$/$^5D_0$-$^7F_1$) as a function of VO_4 content. As described in the main text, the $^5D_0$-$^7F_2$/$^5D_0$-$^7F_1$ intensity ratio increases with higher VO_4 content.

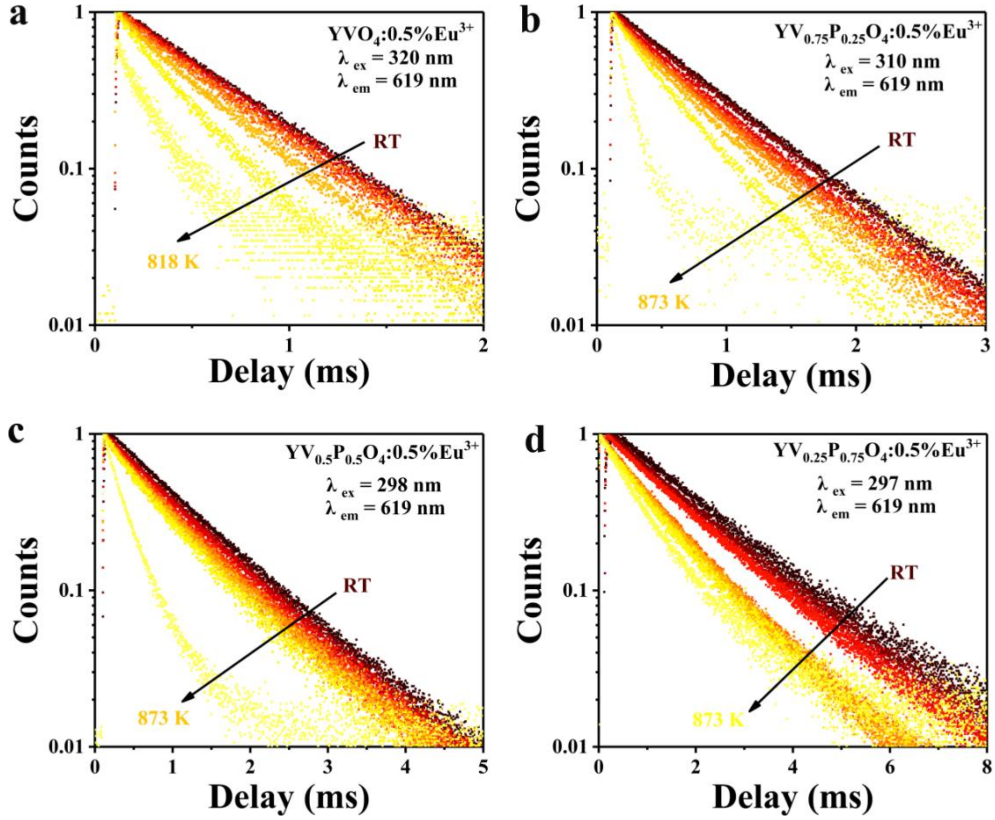

Figure S4. Temperature-dependent luminescence decay curves of the Eu^{3+} emission for single doped $YV_{1-x}P_xO_4$:0.5%Eu^{3+} microcrystals for (a) x=0, (b) x=0.25, (c) x=0.5 and (d) x=0.75. Excitation and emission wavelengths are indicated in the figures.

Table S1. The lifetimes of the Eu^{3+} emission for x varying from 0 to 0.75 at different

temperatures (λ_{em}=619 nm).

T/K	$\tau_{x=0}$/ms	T/K	$\tau_{x=0.25}$/ms	T/K	$\tau_{x=0.5}$/ms	T/K	$\tau_{x=0.75}$/ms
298	0.52	298	0.65	298	0.99	298	1.35
318	0.51	338	0.64	338	0.98	338	1.34
338	0.50	378	0.63	378	0.96	378	1.31
378	0.49	418	0.63	418	0.95	418	1.31
398	0.49	458	0.62	458	0.93	458	1.31
418	0.48	498	0.61	498	0.91	498	1.30
438	0.48	538	0.60	538	0.91	538	1.29
458	0.47	578	0.60	578	0.90	578	1.28
478	0.47	618	0.59	618	0.89	618	1.28
498	0.47	658	0.58	658	0.87	658	1.27
518	0.46	698	0.56	698	0.86	698	1.26
538	0.46	718	0.53	708	0.84	738	1.24
558	0.46	738	0.52	718	0.84	758	1.22
578	0.46	758	0.48	728	0.83	768	1.22
618	0.46	778	0.42	738	0.82	778	1.22
638	0.45	798	0.36	748	0.80	788	1.21
678	0.44	818	0.31	758	0.80	798	1.20
698	0.44	838	0.23	768	0.78	808	1.19
718	0.40	858	0.13	778	0.75	818	1.18
738	0.38	873	0.08	798	0.67	828	1.16
758	0.33			818	0.59	838	1.13
778	0.28			838	0.48	848	1.07
798	0.23			858	0.38	858	1.03
818	0.20			873	0.30	873	0.98
838	0.15						

In **Table S2** the parameters for the fits shown in **Figure 2e**. The temperature

dependence of the decay times was fitted to an exponential function:

$$\boldsymbol{\tau}\,(\mathbf{T}) = \mathbf{C} \times \mathbf{e}^{-\mathbf{T}/\mathbf{A}} + \mathbf{y_0} \qquad \textbf{(1)}$$

where T is the temperature, C is the pre-exponential factor, A is fitting parameter depending on the activation energy and y_0 is a constant and x represents matrix composition.

Table S2. Fitting parameters for modeling the temperature dependence of the luminescence lifetime of Eu^{3+} emission for different values of x in $YV_{1-x}P_xO_4:Eu^{3+}$ using equation (1).

	**x = 0**	**x = 0.25**	**x = 0.5**	**x = 0.75**
**y_0**	0.492	0.634	0.947	1.31
**C**	$-5.71*10^{-5}$	$-5.16*10^{-5}$	$-2.23*10^{-5}$	$-1.98*10^{-6}$
**A**	-103	-93.5	-84.7	-72.5

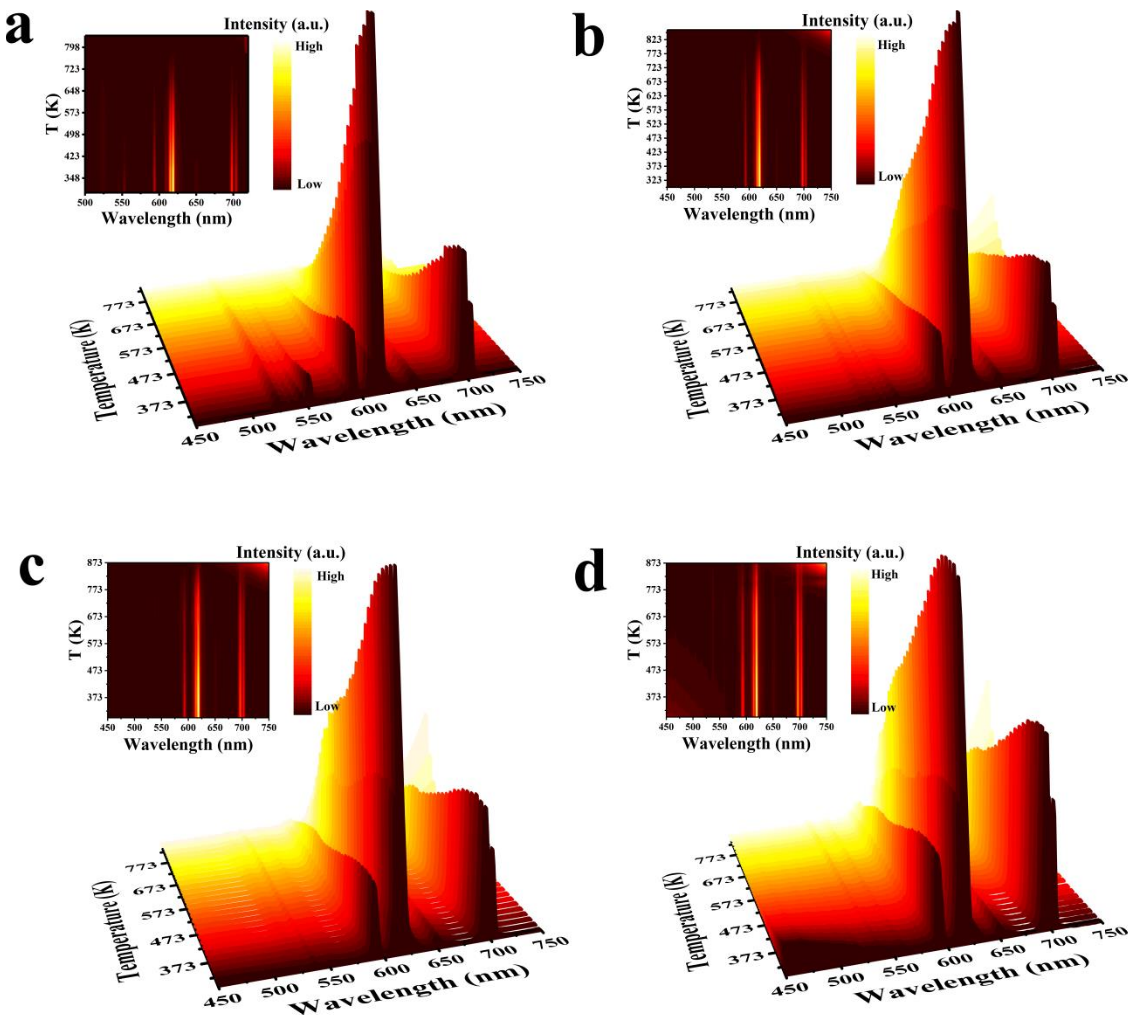

Figure S5. Variable temperature emission spectra of $YV_{1-x}P_xO_4$:0.5%Eu^{3+}, 2%Er^{3+} microcrystals for (a) x=0 (λ_{ex}=310 nm), (b) x=0.25 (λ_{ex}=310 nm), (c) x=0.5 (λ_{ex}=300 nm) and **d.** x=0.75 (λ_{ex}=264 nm).

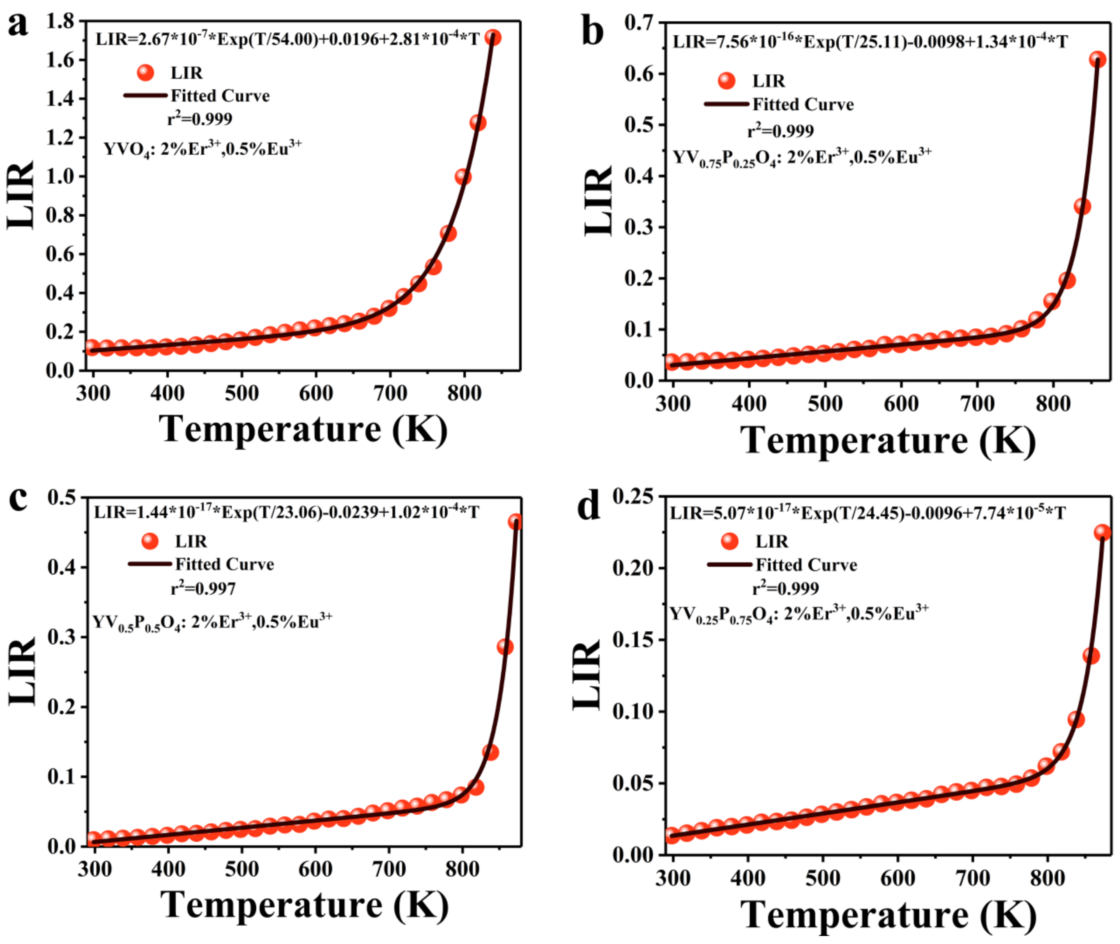

Figure S6. Luminescence intensity ratio of Er^{3+}/Eu^{3+} emission for $YV_{1-x}P_xO_4$:0.5%Eu^{3+}, 2%Er^{3+} microcrystals versus temperature for (a) x=0, (b) x=0.25, (c) x=0.5 and (d) x=0.75.

In **Table S3** the parameters for the fits shown in **Figure S6**. The temperature dependence of the LIR (I_{Er}^{3+}/I_{Eu}^{3+}) was fitted to an empirical formula including the combination of an exponential and linear function:

$$\boldsymbol{LIR = P_1 \times e^{-T/P_2} + P_3 + P_4} \times \mathrm{T} \qquad \textbf{(4)}$$

Where P_1 to P_4 are fitting parameters.

Table S3. Fitting parameters for different *x*-values in equation (4).

	**x = 0**	**x = 0.25**	**x = 0.5**	**x = 0.75**
**P_1**	$2.67*10^{-7}$	$7.56*10^{-16}$	$1.44*10^{-17}$	$5.07*10^{-17}$
**P_2**	-54.0	-25.1	-23.1	-24.5
**P_3**	0.0196	-0.00975	-0.0239	-0.0096
**P_4**	$2.81*10^{-4}$	$1.34*10^{-4}$	$1.02*10^{-4}$	$7.74*10^{-5}$

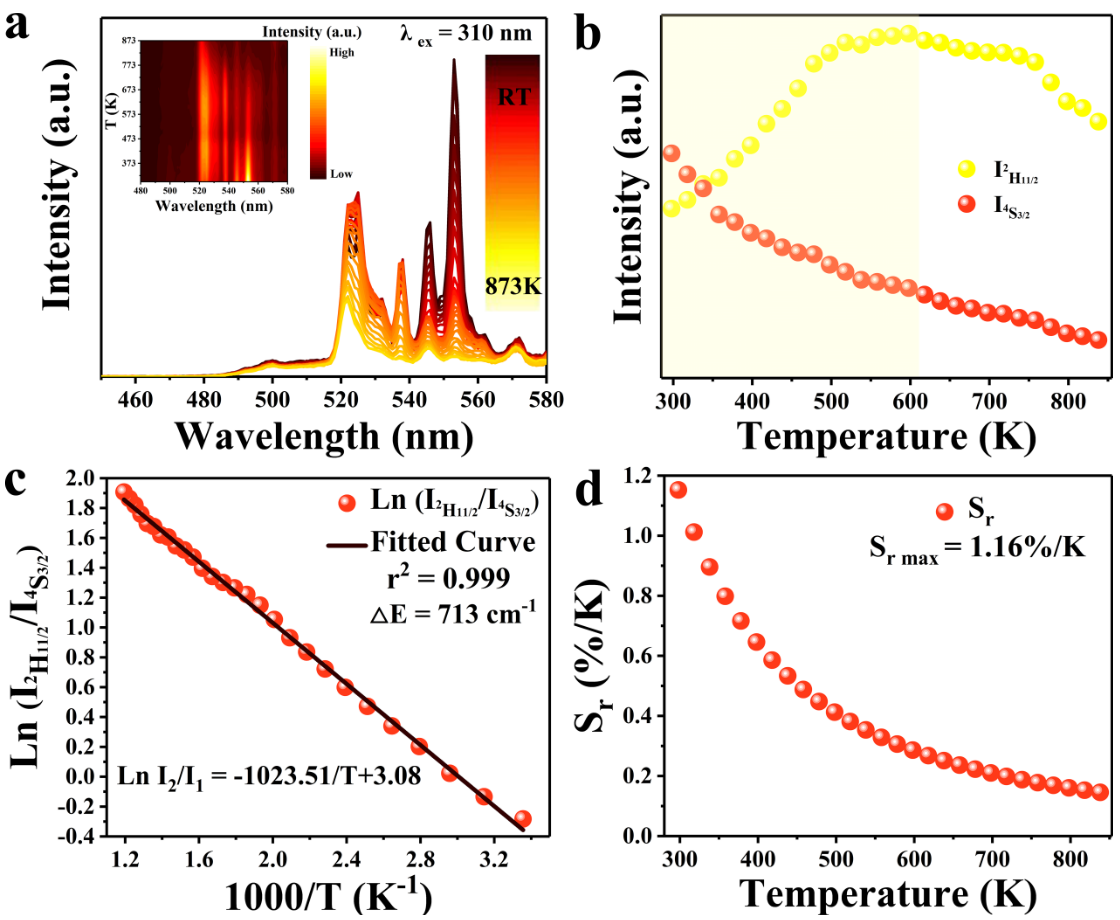

Figure S7. (a) Temperature dependent emission spectra of YVO_4:1%Eu^{3+}, 2%Er^{3+} nanocrystals in the region off Er^{3+} $^2H_{11/2}$-$^4S_{3/2}$ emission under 310 nm excitation. (b) Integrated intensities of $^2H_{11/2}$-$^4I_{15/2}$ and $^4S_{3/2}$-$^4I_{15/2}$ emissions with temperature. (c) Ln ($I_{2H11/2}/I_{4S3/2}$) as function of 1/T. (d) S_r as a function of temperature for temperature sensing based on the $^2H_{11/2}$-$^4S_{3/2}$ LIR in YVO_4:1%Eu^{3+}, 2%Er^{3+} nanoparticles.

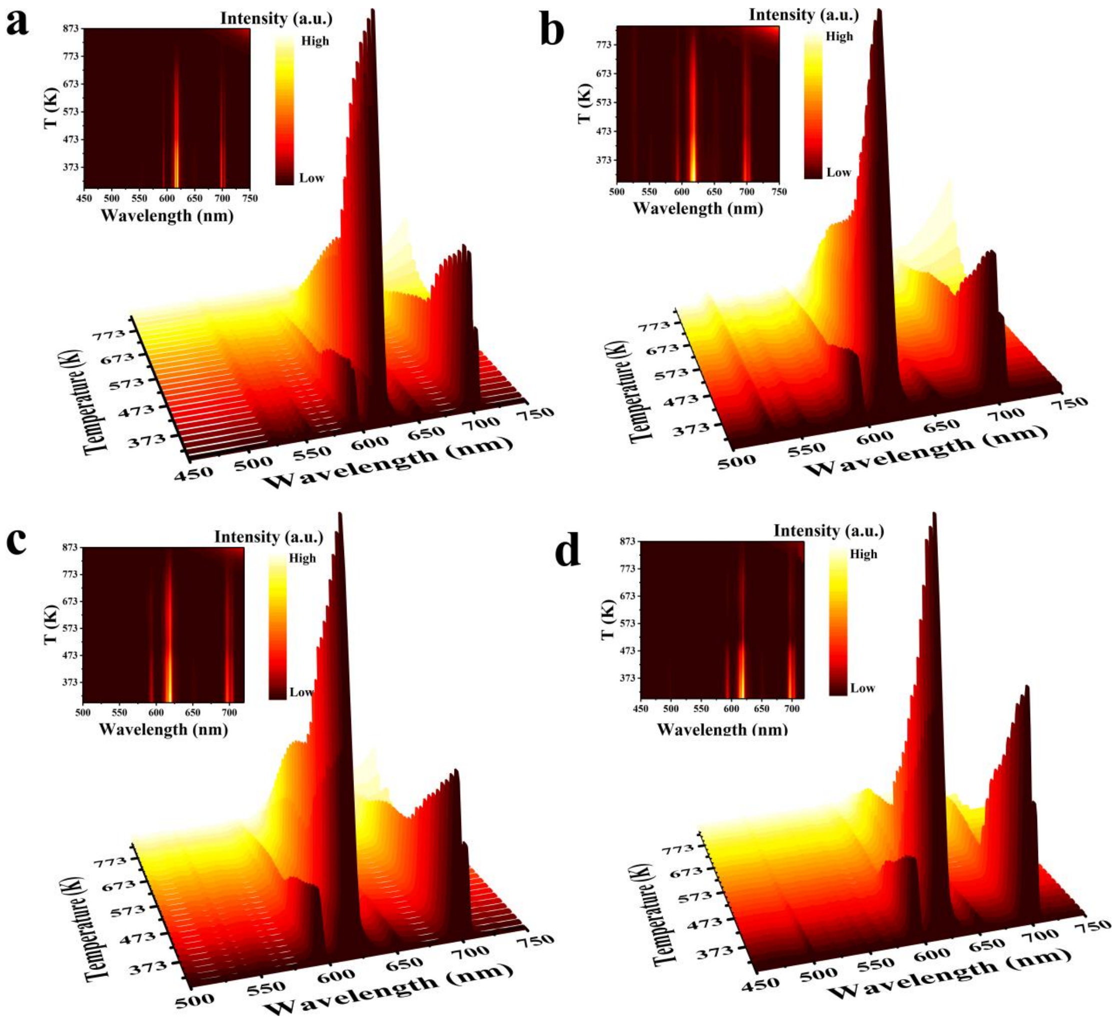

Figure S8. Variable temperature emission spectra of $YV_{1-x}P_xO_4$:2%Er^{3+},1%Eu^{3+} (x=0, 0.25, 0.5 and 0.75) nanocrystals for different P content solid solutions for (a) x=0 (λ_{ex}=310 nm), (b) x=0.25 (λ_{ex}=310 nm), (c) x=0.5 (λ_{ex}=300 nm) and (d) x=0.75 (λ_{ex}=295 nm).

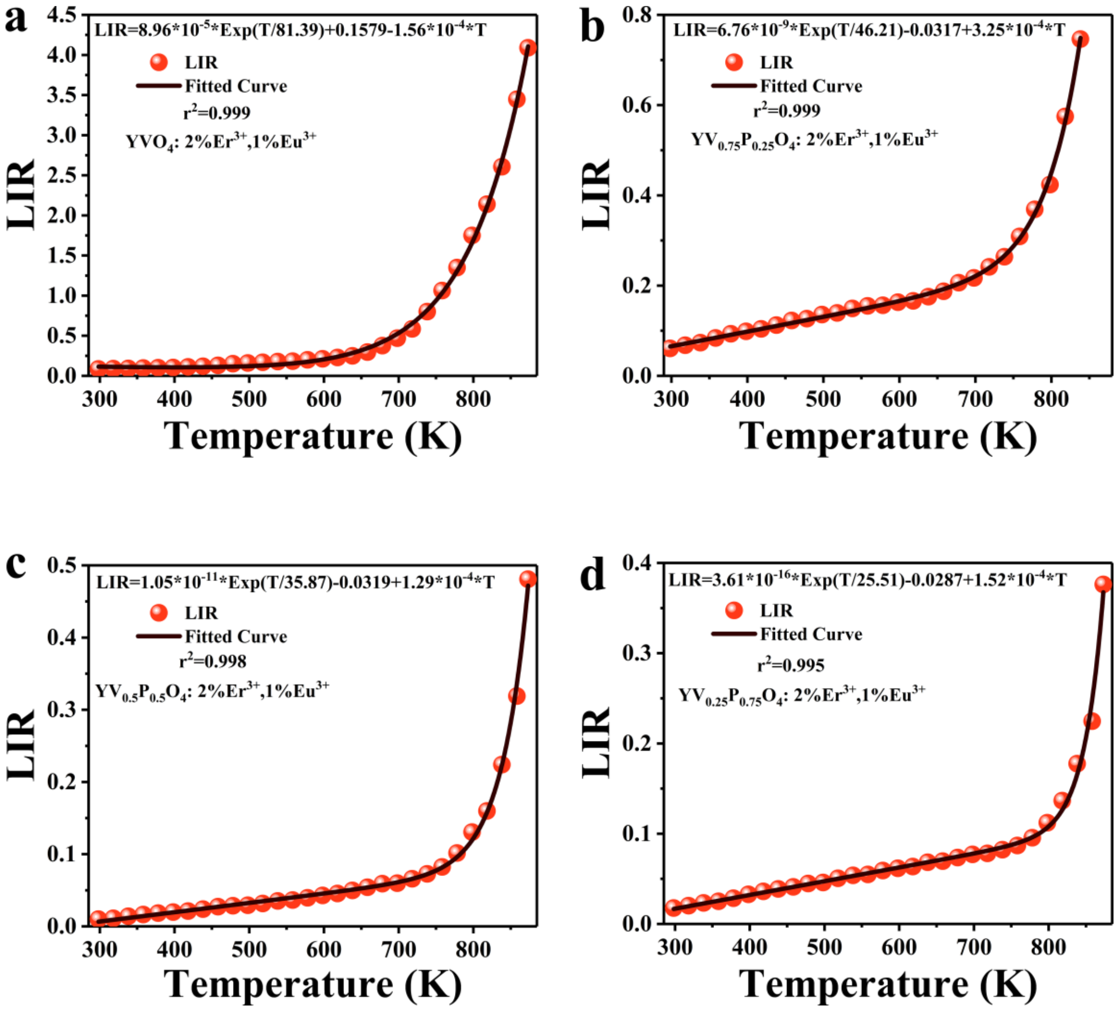

Figure S9. LIR of the Er^{3+}/Eu^{3+} emission intensities for $YV_{1-x}P_xO_4$:2%Er^{3+},1%Eu^{3+} nanocrystals versus temperature for (a) x=0, (b) x=0.25, (c) x=0.5 and (d) x=0.75.

In **Table S4** the parameters for the fits shown in **Figure S9**. The temperature dependence of the LIR ($I_{Er^{3+}}/I_{Eu^{3+}}$) was fitted to an empirical formula including the combination of an exponential and linear function:

$$\boldsymbol{LIR = P_1 \times e^{-T/P_2} + P_3 + P_4} \times \mathrm{T} \qquad \textbf{(5)}$$

Where P_1 to P_4 are fitting parameters. This empirical fit is similar to the function used for the microcrystalline system.

Table S4. Fitting parameters for different x-values in equation (5).

	**x = 0**	**x = 0.25**	**x = 0.5**	**x = 0.75**
**P_1**	$8.96*10^{-5}$	$6.76*10^{-9}$	$1.05*10^{-11}$	$3.61*10^{-16}$
**P_2**	-81.4	-46.2	-35.9	-25.5
**P_3**	0.158	-0.0317	-0.0319	-0.0287
**P_4**	$-1.56*10^{-4}$	$3.25*10^{-4}$	$1.29*10^{-4}$	$1.52*10^{-4}$

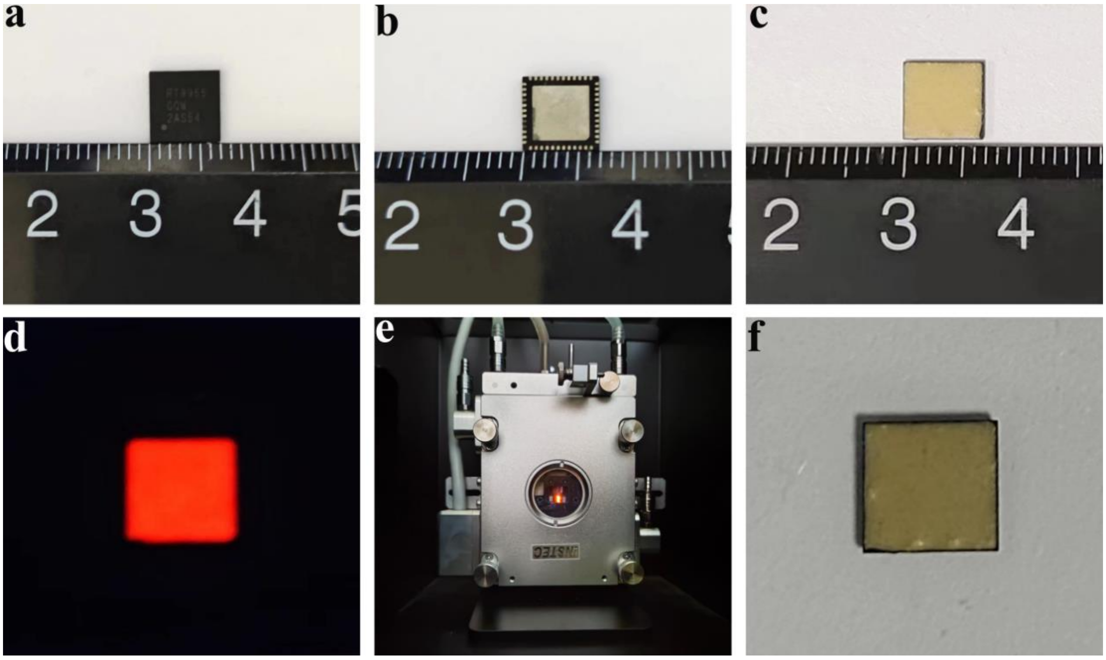

Figure S10. (a-b) Photographs of the front and back sides of the RT9955 chip without the T-probe covering. (c) Photograph of the chip surface after packaging with luminescent thermometer. (d) Photograph of the chip with packaged luminescent thermometer under UV light. (e) Photograph of the chip with packaged luminescent thermometer placed in a variable temperature device and excited at 310nm. (f) Photograph of the chip cooled to room temperature after variable temperature treatment at RT-473K. The temperature probes are thoroughly mixed with the adhesive (powder-to-adhesive ratio of 1:5) and coated onto the surface of the chip. Subsequently, the chip, coated with the temperature probes, is placed in a temperature control device connected to a computer to set and monitor real-time temperature. Temperature dependent spectra are obtained by connecting the system to a spectrometer. Each temperature is calculated using the calibration curve described in the main text and compared with the real-time temperature, from which the temperature error is determined. These in situ temperature sensing experiments provide a clear demonstration of the accuracy and systematic errors of our temperature sensors applied to in situ temperature measurement on chip. After heating from RT to 473K and then cooling back to RT, the luminescence thermometers encapsulated on the chip surface showed no significant changes, indicating the stability of this approach. The slight blackening of the color is due to the mild decomposition or oxidation of the adhesive at 473K.

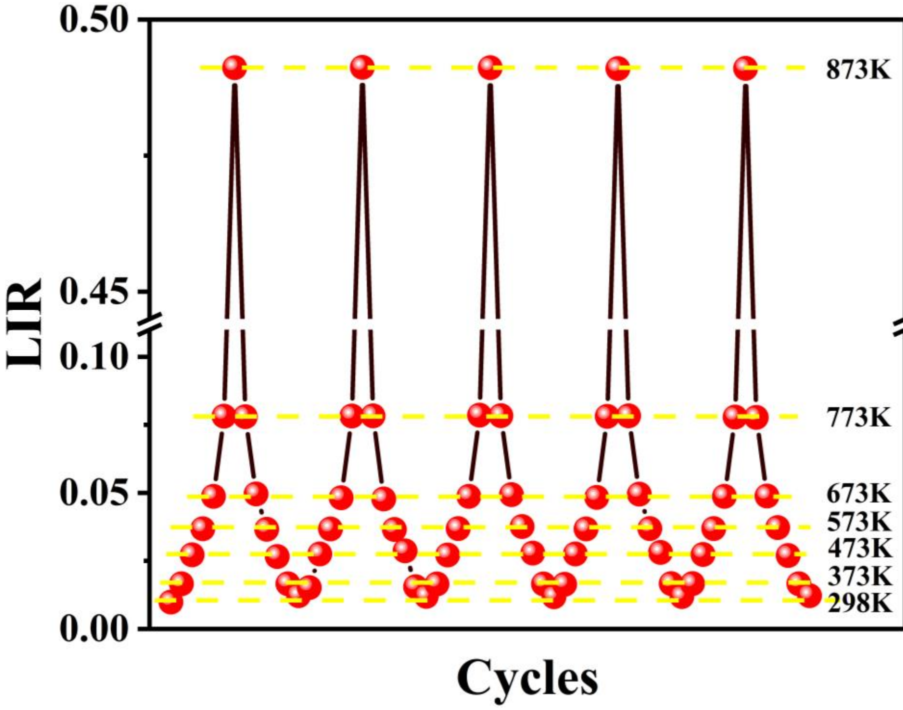

Figure S11. Luminescence intensity ratios for the Er^{3+}/Eu^{3+} emission intensities in $YV_{0.5}P_{0.5}O_4$:0.5%Eu^{3+}, 2%Er^{3+} microcrystals for five consecutive heating and cooling cycles from 298K to 873K with 100K intervals. The reproducibility observed in the various heating and cooling cycles shows that these temperature probes have good durability and stability.

Table S5. ICP-OES analysis of a number of $YV_{1-x}P_xO_4$:0.5%Eu^{3+}, 2%Er^{3+} microcrystals (M) and $YV_{1-x}P_xO_4$:2%Er^{3+}, 1%Eu^{3+} nanocrystals (N) for x=0, 0.25 and 0.5. The results show that the actual composition derived from the ICP-OES data for the materials is close to the intended composition based on the ratios of the starting materials.

	**Elemental content by ICP-OES Mass fraction (W/%)**					**Actual composition**
	**Y**	**V**	**P**	**Eu**	**Er**	
**M with x = 0**	39.5	23.3	/	0.370	1.54	$Y_{0.975}Eu_{0.005}Er_{0.020}VO_4$
**M with x = 0.25**	33.2	15.5	3.29	0.276	1.17	$Y_{0.976}Eu_{0.005}Er_{0.019}V_{0.74}P_{0.26}O_4$
**M with x=0.5**	19.4	6.73	3.82	0.209	0.772	$Y_{0.974}Eu_{0.006}Er_{0.020}V_{0.52}P_{0.48}O_4$
**N with x = 0**	36.1	23.2	/	0.805	1.52	$Y_{0.966}Eu_{0.012}Er_{0.022}VO_4$
**N with x = 0.25**	34.6	17.1	3.54	0.821	1.53	$Y_{0.966}Eu_{0.012}Er_{0.022}V_{0.75}P_{0.25}O_4$
**N with x=0.5**	34.9	11.1	6.48	0.920	1.51	$Y_{0.963}Eu_{0.015}Er_{0.022}V_{0.51}P_{0.49}O_4$

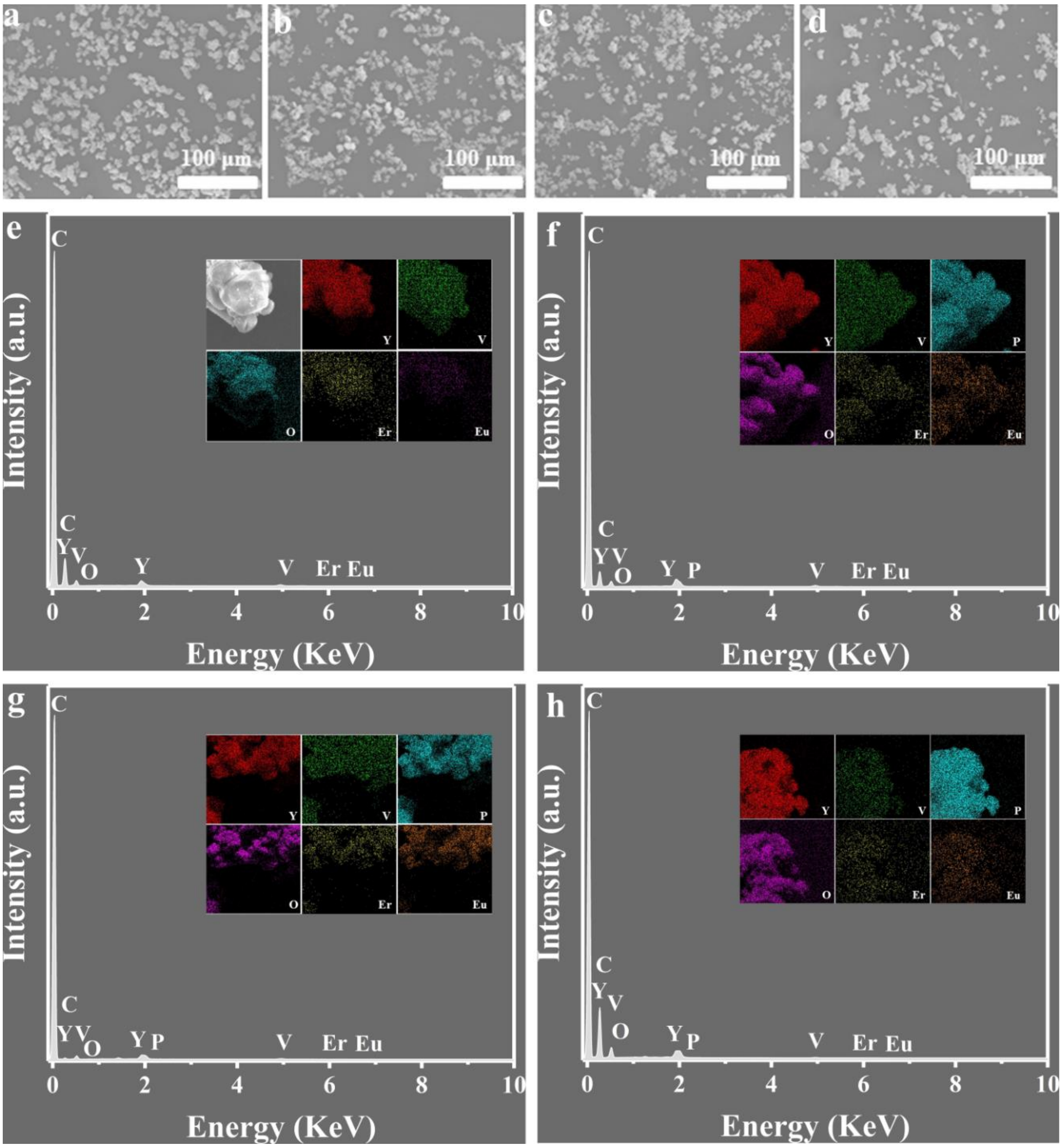

Figure S12. SEM images of $YV_{1-x}P_xO_4$:0.5%Eu^{3+}, 2%Er^{3+} microcrystals for (a) x=0, (b) x=0.25, (c) x=0.5 and (d) x=0.75. EDX spectra of $YV_{1-x}P_xO_4$:0.5%Eu^{3+}, 2%Er^{3+} microcrystals for (e) x=0, (f) x=0.25, (g) x=0.5 and (h) x=0.75. The insets show SEM mapping of Y, V, P, O, Eu and Er, respectively. All elements appear evenly distributed over the particles. Moreover, no peaks of impurity elements appear (the appearance of C is attributed to the carbon film).
